# Drug interventions for acute treatment of pediatric migraine: a systematic review and network meta-analysis

**DOI:** 10.3389/fphar.2026.1851994

**Published:** 2026-07-15

**Authors:** Yi Cheng, Jiaqi Li, Lei Liu, Guanglu Li

**Affiliations:** 1 College of Traditional Chinese Medicine, Hebei University, Baoding, China; 2 Department of Neurology, China-Japan Friendship Hospital, Beijing, China; 3 Graduate School of Beijing University of Chinese Medicine, Beijing, China; 4 Qingdao Municipal Hospital, Qingdao, China

**Keywords:** adolescents, children, migraine, network meta-analysis, systematic review

## Abstract

**Background:**

Migraine in children and adolescents not only impacts academic pursuits and family life but also have secondary psychological effects. Determining the role of acute medication for migraine treatment in this population can reduce the burden associated with migraine. This network meta-analysis aimed to identify the relative efficacy and safety of acute migraine drug in children and adolescents migraine populations.

**Methods:**

The Cochrane Register of Controlled Trials and MEDLINE via PubMed and Embase databases were searched from inception to August 2025, only published studies in English. Double-blind randomized clinical trials evaluating the currently available acute treatments for childhood and adolescent migraines were included. The primary efficacy endpoint was pain freedom at 2 hours. Secondary efficacy endpoints included the proportion of participants with pain relief at 2 hours, pain freedom from two to 24 h, and the proportion using rescue drugs after 2 hours and up to 24 h. Adverse events (AEs) were also evaluated.

**Results:**

The analysis included 30 studies (involving 8,914 participants and 13 pharmacological interventions). All treatments included demonstrated higher odds ratios (ORs) compared with the placebo for pain freedom at 2 hours. Dihydroergotamine was associated with the highest ORs, but its confidence interval included null values. Sumatriptan/naproxen sodium, ibuprofen, zolmitriptan nasal spray, sumatriptan nasal spray, and rizatriptan showed statistical significance. Sumatriptan/naproxen sodium yielded the highest odds (OR: 2.91, 95% CI: 1.87–4.53), followed by ibuprofen (OR: 2.88, 95% CI: 1.47–5.64), and rizatriptan showed the lowest (OR: 1.51, 95% CI: 1.23–1.86). Only sumatriptan/naproxen sodium was associated with a significantly higher OR compared with placebo for pain freedom from two to 24 h (OR: 2.31, 95% CI: 1.31–4.07). Ibuprofen exhibited the highest effect size for pain relief at 2 hours (OR: 3.21, 95% CI: 1.10–9.34). None of the included drugs was found to reduce the use of rescue drugs from two to 24 h. Zolmitriptan was associated with the highest risk of AEs among all treatments. Acetaminophen appears to have the lowest risk of adverse events, comparable to that of a placebo.

**Conclusion:**

Ibuprofen can effectively relieve symptoms, characterized by a favorable benefit-risk profile. Sumatriptan and zolmitriptan nasal sprays also exhibited robust efficacy, specifically among populations with prominent nausea and vomiting. Sumatriptan/naproxen sodium merits consideration, especially in patients exhibiting an inadequate response to monotherapy. Dihydroergotamine demonstrated potential benefits in refractory and chronic migraine; however, high-quality studies are warranted to validate these findings.

## Introduction

Migraine is a common, disabling primary headache disorder in the pediatric population (Ashina, 2020; Ferrari et al., 2022), affecting 11% of children and adolescents (Onofri et al., 2023). Compared to adults, pediatric and adolescent migraines usually present with atypical features, such as shorter attack duration, more frequent attacks, usually bilateral frontal-temporal headaches, and more widespread gastrointestinal symptoms (Patniyot and Qubty, 2020; Khan et al., 2025). It may also present with episodic syndromes associated with migraine, such as periodic vomiting, benign paroxysmal torticollis, and benign paroxysmal vertigo (Diener et al., 2008). Headaches can limit social events and physical activities, increasing the risk of absenteeism and dropout (Arruda and Bigal, 2012; Falla et al., 2022). Migraine can cause negative affective states (such as anxiety, depression, and anger) and decreased quality of life ratings in children and adolescents (Kohandel et al., 2024). Effective management of migraine in childhood and adolescence can prevent migraine-related disability and improve long-term outcomes as patients transition into adulthood.

Management of migraine in children is complex and multifaceted. International clinical guidelines generally endorse non-steroidal anti-inflammatory drugs (NSAIDs) as initial drug treatment for both children and adolescents with acute migraine attacks (Abu-Arafeh and Howells, 2024). Triptans have proved effective in treating moderate to severe headaches in adolescents. The combination of sumatriptan and naproxen sodium shows promise in the treatment of adolescent migraine (Lai et al., 2017; Derosier et al., 2012). The US Food and Drug Administration (FDA) has only approved rizatriptan for the acute management of migraine in children older than 6 years, and sumatriptan, zolmitriptan, and almotriptan for adolescents older than 12 years (Gibler et al., 2022).

Due to the complexity of pediatric migraine and the paucity of high-quality direct comparative studies, there is insufficient evidence to establish a definitive treatment hierarchy within these pharmacological classes. Furthermore, the substantial placebo effect complicates the assessment of treatment efficacy in this population. In the absence of direct comparative data, network meta-analysis (NMA) provides a method for conducting multiple comparisons simultaneously through a single analysis, thereby offering evidence for informed clinical decision-making. Here, we conducted an NMA to systematically evaluate the relative efficacy, safety, and tolerability of existing medications for the acute treatment of migraine in children and adolescents. The findings of this NMA will inform clinicians in the management of childhood and adolescent migraine.

## Methods

### General guidelines applied

**FIGURE 1:**
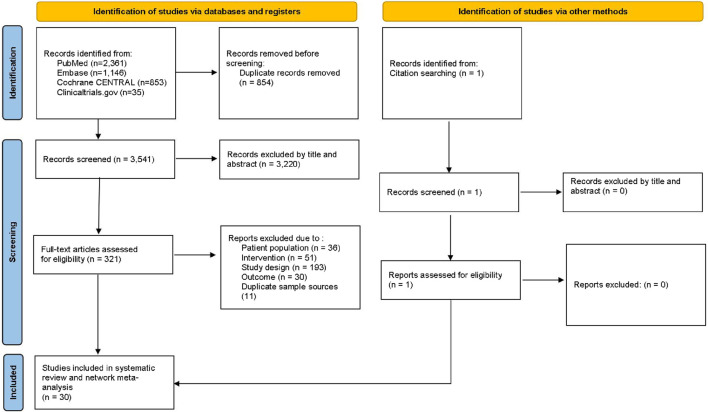
Flow Chart of the network meta-analysis procedure.

This review followed the recommendations of the NMA guidelines for Preferred Reporting Items for Systematic Reviews and Meta-Analyses (PRISMA-NMA) reporting guideline checklist (Hutton et al., 2015). The completed PRISMA-NMA checklist is provided in Supplementary Appendix 1. The PRISMA flowchart of the screened studies is presented in Figure 1 and was registered in the International Prospective Register of Systematic Reviews (PROSPERO: CRD42024582523).

### Literature search

A systematic literature search was conducted across the Cochrane Register of Controlled Trials and the MEDLINE database via PubMed, as well as the Embase database. The search was limited to published English-language articles covering the period from inception to August 2025. Individuals under 12 years were defined as children, and those aged 12–17 years as adolescents. Detailed search strategies tailored to each database are provided in Supplementary Appendix 2. We also examined the clinicaltrials.gov registry to identify ongoing trials. To further supplement our search, we screened reference lists from existing pairwise meta-analyses and relevant review articles (Richer et al., 2016; Chanchlani et al., 2023). The entire literature search process was conducted independently by two authors (Yi Cheng, Jiaqi Li).

### Eligibility criteria

To increase the reliability of the NMA, studies needed to meet the following PICOS criteria.

Population (P): The population comprised outpatients and individuals younger than 18 years with episodic or chronic migraine, with or without aura, as defined by the International Classification of Headache Disorders (ICHD) or the version of ICHD in use during the study period.

Intervention (I): We included studies of pharmacological drugs for the acute treatment of migraine in children and adolescents. Only drugs licensed for migraine or headache were considered eligible if they were recommended by at least one of the regulatory bodies internationally (Hassan et al., 2026). There were no restrictions on the dose, formulation, dosing regimen, or timing of the study medication. Clinical guidelines discourage opiates for migraine due to their limited efficacy, considerable adverse effects, and the risk of dependence; therefore, they were excluded from our analysis. Furthermore, studies conducted in emergency department settings were also excluded, as the patient population in this context typically represents a subgroup experiencing particularly severe or atypical episodes.

Comparator (C): We included head-to-head comparisons of two pharmacological drugs with each other or a placebo.

Outcome (O): The study had to report at least one clinical outcome indicator that we were concerned about.

Study design (S): We only included RCTs with human participants that were fully published in English.

### Primary and secondary outcome measures

We referred to the guidelines and available indicators from the included studies, selecting pain freedom (defined as the absence of pain based on a 4-point global scale) at 2 hours before using any rescue medication as the primary efficacy outcome (Diener et al., 2019). Secondary efficacy endpoints included the proportion of participants experiencing pain relief at 2 hours post-dose, pain freedom from two to 24 h, and the proportion of participants using rescue drugs after 2 hours and up to 24 h. The outcomes for safety and tolerability were adverse events (AEs) within 48 h post-dose.

### Study screening and data collection

All returned data extraction processes followed a standardized form. After the initial search, one pair of reviewers (Yi Cheng, Jiaqi L) independently performed the following operations: removing duplicates, reviewing the titles and abstracts of all identified citations for primary screening, and retrieving and screening full-text papers according to eligibility criteria. Disagreements were resolved through discussion, and if necessary, by a third senior author. For each study meeting the eligibility criteria, data were independently extracted by two reviewers utilizing a pre-defined Excel template. All extracted data were verified for consistency and accuracy by a third reviewer. Any discrepancies in evaluating these data were resolved by discussion or consultation. The third author evaluated the data that could not be resolved until a consensus was reached.

### Quality assessment and risk of bias

The quality of the included studies was assessed using a standardized table based on the Cochrane Risk of Bias tool version 2 (RoB2) (Sterne et al., 2019) by the two primary reviewers (Guanglu Li, Yi Cheng) independently. Confidence in Network Meta-Analysis (CINeMA) (Nikolakopoulou et al., 2020) was used to assess the quality of evidence for the outcome indicators of the NMA. CINeMA is an approach for determining confidence in the results of an NMA broadly based on the Grading of Recommendations Assessment, Development, and Evaluation (GRADE). We utilized a freely available, user-friendly online CINeMA web application (Papakonstantinou et al., 2020) to assess confidence in the results from the NMA. Finally, comparative-adjusted funnel plots and Egger regression (Chaimani et al., 2013) were used to determine potential minor study effects and publication bias.

### Statistical analysis

All network meta-analyses were performed using R software. Due to the heterogeneity and relatively limited sample size of the included studies, we apply frequentist random-effects models to estimate odds ratios (ORs) with 95% confidence intervals (CIs) for each primary and secondary outcome (Rucker, 2012; Rücker and Schwarzer, 2014). We used the frequentist theory model with the mvmeta command to compare effect sizes across studies involving the same treatment regimens. All comparisons were two-tailed, and a p-value cut-off point of 0.05 denoted statistical significance (Hi et al., 2003). Heterogeneity among the included studies was assessed using the tau value and the I^2^ statistic.

The validity of NMA rests on three core assumptions. First, the transitivity assumption requires that relative treatment effects are consistent across studies comparing different interventions. To uphold this, We included only pharmacologic agents approved by international regulatory authorities for the treatment of migraine in children and adolescents. Given the limited number of available trials in this population, we did not impose restrictions on dose, formulation, dosing regimen, or timing of initial administration. To account for potential effect modification, separate NMA models were fitted for oral formulations. Second, the similarity assumption requires clinical and methodological homogeneity across included studies. We therefore included only RCTs. Although the number of eligible studies precluded stratifying by age subgroups in the primary analysis, subgroup analyses were conducted separately for children and adolescents to explore potential differences in treatment effects. Third, the consistency assumption implies agreement between direct and indirect evidence sources. We conducted a statistical evaluation of inconsistency. A loop-specific approach and the node-splitting method (Dias et al., 2010) were employed to examine local inconsistency within the model, while the design-by-treatment interaction model was applied to assess global inconsistency across the entire NMA.

One of the advantages of NMA is its ability to rank competing interventions. We employed the SUCRA proposed by Salanti et al. to present the NMA results (Salanti et al., 2011). It is calculated by averaging cumulative rank probabilities and transforms the mean rank of a treatment to a value between 0 and 1. The advantage of SUCRA is that it has a common range from 0 to 1, facilitating consistent interpretation of different NMAs (Rosenberger et al., 2021). SUCRA values of 1 indicate that the treatment might be the best and 0 the worst. However, SUCRA may be misleading when networks are sparse, confidence intervals are wide, and certainty of evidence is low (Caldwell et al., 2005).

### Additional analyses

We excluded high-risk-of-bias studies from the primary efficacy endpoint network to assess the robustness of the results. We also constructed separate networks for pediatric and adolescent migraine patients to obtain extra findings. Additionally, the relative efficacy of oral pharmacological agents was assessed separately.

## Results

### Search results and study characteristics

Through the initial literature search phase, a total of 4,395 database records were identified. An additional literature search was conducted through citation searching. After removing duplicate publications and conducting abstract screening, 321 studies underwent full-text screening. Ultimately, based on the eligibility criteria, 30 studies (involving 8,914 participants and 13 pharmacological interventions) that met the inclusion criteria were included in the NMA.

**FIGURE 2:**
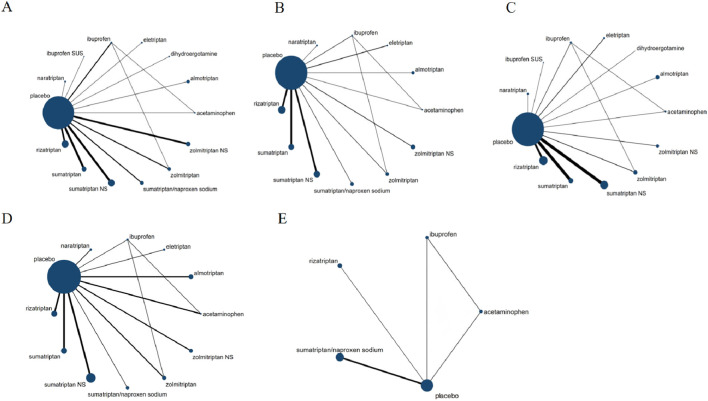
Network plot for primary outcomes and secondary outcomes The lines between nodes represent direct comparisons in various trials, and each circle’s size is proportional to the population involved in each specific treatment. The thickness of the lines is proportional to the number of trials connected to the network. NS, nasal spray; SUS, suspension. **(A)** Pain freedom at 2 hours. **(B)** Adverse events. **(C)** Pain relief at 2 hours. **(D)** Use of rescue drugs from 2 to 24 hours. **(E)** Pain freedom from 2 to 24 hours.

We included the primary medications commonly used for the acute treatment of migraine, including triptans (sumatriptan, zolmitriptan, eletriptan, rizatriptan, almotriptan, and naratriptan), NSAIDs (ibuprofen, naproxen sodium, and acetaminophen), and ergot derivatives. The network plot of treatment comparisons is shown in Figure 2. All included studies were published between 1997 and 2022 and adhered to the clinical guidelines in effect at the time of their execution. A total of 8,914 randomized children and adolescents received pharmacological treatment. Twenty-eight studies were two-group randomized clinical trials, while two consisted of three-group studies. Eight studies consisted of mixed populations involving both children and adolescents. Among the remaining 22 studies, the majority (77.3%, 17/22) focused on adolescent migraine, with only five studies specifically addressing childhood migraine. Eight studies reported on the use of prophylactic medication, with only three explicitly prohibiting the use of such drugs before the trial. The baseline demographic characteristics and headache features of the 30 studies included in the NMA are given in Supplementary Table 1. We labelled the heterogeneity statistic I^2^ of each outcome in the upper right of the forest plot and only detected substantial heterogeneity in the endpoint of AEs (I^2^ = 59.4%).

### Efficacy

#### Primary efficacy outcome: pain freedom at 2 h

**FIGURE 3:**
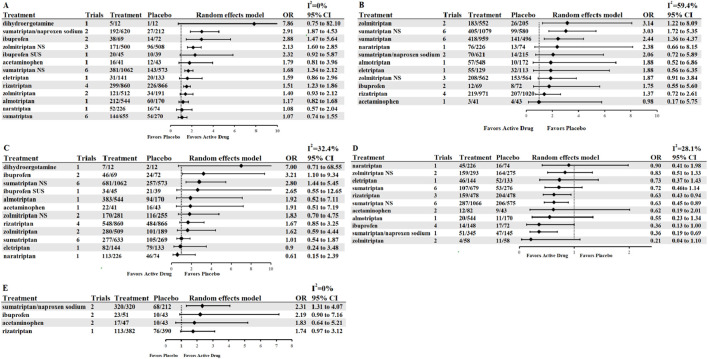
Forest plot for primary outcomes and secondary outcomes. The forest plot was based on a random-effects model. The data behind the drug names indicate the following: Trials, number of trials examining the current active drug; Treatment/Placebo, number of patients with events/number of patients in which the drug was examined in these trials; OR, odds ratio; CI, confidence interval. 95% CI that did not contain one and a p-value cutoff point of 0.05 was considered statistically significant. I^2^ values of less than 50% indicate that heterogeneity may not be significant; a value higher than 50% may represent substantial heterogeneity. For the effectiveness endpoint, results to the left of 1 favor placebo, to the right favor intervention, result in adverse events was the opposite. NS, nasal spray; SUS, suspension. **(A)** Pain freedom at 2 hours. **(B)** Adverse events. **(C)** Pain relief at 2 hours. **(D)** Use of rescue drugs from 2 to 24 hours. **(E)** Pain freedom from 2 to 24 hours.

The network diagram for the primary outcome of pain freedom at 2 h included 30 studies and 13 intervention nodes (Figures 2A, 3A). All specific pharmacologic treatments included demonstrated higher odds ratios compared with placebo, but only sumatriptan/naproxen sodium, ibuprofen, zolmitriptan nasal spray, sumatriptan nasal spray, and rizatriptan showed statistical significance. Sumatriptan/naproxen sodium yielded the highest odds (OR: 2.91, 95% CI: 1.87–4.53), followed by ibuprofen (OR: 2.88, 95% CI: 1.47–5.64), and rizatriptan showed the lowest (OR: 1.51, 95% CI: 1.23–1.86) (Table 1; Figure 3A). Dihydroergotamine showed the highest point estimate among all treatments, but its confidence interval contained the null value.

**TABLE 1:** League table of pain freedom at 2 h.

Acetaminophen	1.58 (0.58, 4.25)	​	​	0.61 (0.28, 1.31)	​	​	​	​	​	​	​	​	1.65 (0.62, 4.41)
1.53 (0.64, 3.65)	**Almotriptan**	​	​	​	​	​	​	​	​	​	​	​	1.17 (0.70, 1.94)
0.23 (0.02, 2.71)	0.15 (0.01, 1.60)	**Dihydroergotamine**	​	​	​	​	​	​	​	​	​	​	7.85 (0.73, 84.37)
1.12 (0.41, 3.08)	0.74 (0.36, 1.51)	4.93 (0.44, 55.89)	**Eletriptan**	​	​	​	​	​	​	​	​	​	1.59 (0.77, 3.25)
0.62 (0.31, 1.25)	0.41 (0.19, 0.87)	2.72 (0.24, 31.28)	0.55 (0.22, 1.38)	**Ibuprofen**	​	​	​	​	​	​	*1.15 (0.38, 3.42)	​	**5.28 (2.26, 12.31)**
0.77 (0.23, 2.62)	0.50 (0.19, 1.37)	3.39 (0.27, 42.24)	0.69 (0.22, 2.10)	1.24 (0.40, 3.91)	**Ibuprofen SUS**	​	​	​	​	​	​	​	2.32 (0.85, 6.27)
1.65 (0.60, 4.56)	1.08 (0.52, 2.24)	7.25 (0.64, 82.44)	1.47 (0.61, 3.57)	**2.66 (1.06, 6.70)**	2.14 (0.70, 6.59)	**Naratriptan**	​	​	​	​	​	​	1.08 (0.52, 2.24)
1.18 (0.52, 2.69)	0.78 (0.51, 1.17)	5.20 (0.49, 54.85)	1.05 (0.55, 2.03)	1.91 (0.95, 3.85)	1.54 (0.59, 3.98)	0.72 (0.37, 1.40)	**Rizatriptan**	​	​	​	​	​	**1.52 (1.15, 2.03)**
1.67 (0.70, 4.02)	1.10 (0.65, 1.84)	7.35 (0.68, 79.11)	1.49 (0.72, 3.07)	**2.70 (1.25, 5.81)**	2.17 (0.80, 5.90)	1.01 (0.49, 2.11)	1.41 (0.92, 2.16)	**Sumatriptan**	​	​	​	​	1.07 (0.71, 1.61)
0.61 (0.25, 1.52)	0.40 (0.23, 0.71)	2.70 (0.25, 29.40)	0.55 (0.26, 1.17)	0.99 (0.44, 2.21)	0.80 (0.29, 2.23)	0.37 (0.17, 0.81)	0.52 (0.32, 0.85)	0.37 (0.21, 0.65)	**Sumatriptan/naproxen sodium**	​	​	​	**2.90 (1.74, 4.83)**
1.06 (0.46, 2.43)	0.70 (0.45, 1.07)	4.66 (0.44, 49.31)	0.95 (0.49, 1.83)	1.71 (0.84, 3.48)	1.38 (0.53, 3.59)	0.64 (0.33, 1.26)	0.90 (0.66, 1.22)	0.63 (0.41, 0.98)	**1.73 (1.05, 2.84)**	**Sumatriptan NS**	​	​	**1.72 (1.28, 2.32)**
1.28 (0.55, 2.98)	0.84 (0.48, 1.44)	5.61 (0.52, 60.73)	1.14 (0.54, 2.39)	**2.06 (1.02, 4.17)**	1.66 (0.60, 4.57)	0.77 (0.36, 1.65)	1.08 (0.68, 1.71)	0.76 (0.44, 1.33)	**2.08 (1.13, 3.80)**	1.20 (0.75, 1.93)	**Zolmitriptan**	​	1.48 (0.86, 2.53)
0.84 (0.36, 1.95)	0.55 (0.35, 0.87)	3.68 (0.35, 39.20)	0.75 (0.38, 1.48)	1.35 (0.65, 2.81)	1.09 (0.41, 2.88)	0.51 (0.25, 1.02)	0.71 (0.50, 1.01)	0.50 (0.31, 0.80)	1.36 (0.80, 2.31)	0.79 (0.55, 1.14)	0.66 (0.40, 1.09)	**Zolmitriptan NS**	**2.13 (1.49, 3.05)**
1.79 (0.81, 3.96)	1.17 (0.82, 1.68)	7.86 (0.75, 82.10)	1.59 (0.86, 2.96)	**2.88 (1.47, 5.64)**	2.32 (0.92, 5.87)	1.08 (0.57, 2.04)	**1.51 (1.23, 1.86)**	1.07 (0.74, 1.55)	**2.91 (1.87, 4.53)**	**1.68 (1.34, 2.12)**	1.40 (0.93, 2.12)	**2.13 (1.60, 2.85)**	**Placebo**

Odds ratios (OR) with 95% confidence interval (CI) (OR, of > 1 indicated that the treatment specified in the row got more improvement than that specified in the column), 0 < OR < 1, the opposite. For the network meta-analysis, OR, of > 1 indicated that the treatment specified in the column got better improvement than that specified in the row, 0 < OR < 1, the opposite. 95% CI, that did not contain one was considered to have a statistical difference. Bold results indicated statistical significance. Marked with * indicated a significant difference between direct and mixed comparisons. ^ indicated a significant difference between the basic model and sensitivity analysis. NS, nasal spray; SUS, suspension.

In the base model, sumatriptan/naproxen sodium demonstrated better efficacy than sumatriptan NS (OR: 1.73, 95% CI: 1.05–2.84) and zolmitriptan (OR: 2.08, 95% CI: 1.13–3.80). Ibuprofen showed statistically superior efficacy compared to zolmitriptan in both direct (OR, 1.15; 95% CI, 0.38–3.42) and mixed comparisons (OR, 2.06; 95% CI, 1.02–4.17). Furthermore, the base model indicated that ibuprofen had higher odds ratios than naratriptan (OR: 2.66, 95% CI: 1.06–6.70) and sumatriptan (OR: 2.70, 95% CI: 1.25–5.81). According to the SUCRA rankings (Supplementary Table 2), dihydroergotamine was ranked as the most effective treatment, followed by sumatriptan/naproxen and ibuprofen. The results of the sensitivity analysis in the Supplementary Material (Supplementary Table 10), excluding seven studies with a high risk of bias to assess the reliability of the combined effect sizes, were consistent with those of the base model.

#### Secondary efficacy outcome: pain relief at 2 h

The network diagrams are shown in Figures 2C, 3C. In brief, the network diagrams display 28 studies and 12 intervention nodes. All active drugs showed higher odds ratios than placebo, but only ibuprofen and sumatriptan NS were statistically significant. Mixed comparisons between active agents did not reveal any statistically significant findings. The SUCRA rankings indicated that dihydroergotamine was the most effective treatment for pain relief at 2 h.

#### Secondary efficacy outcome: use of rescue drugs from 2 to 24 h

The network diagram shows 29 studies and 11 intervention nodes (Figure 2D). For the outcome of rescue medication use within 2–24 h, no statistically significant results were found. Zolmitriptan and sumatriptan/naproxen sodium appear to be the drugs requiring the least use of rescue medication from 2 to 24 h, yet the confidence intervals include null values.

#### Secondary efficacy outcome: pain freedom from two to 24 h

A total of 7 studies and 4 treatment nodes were included in the analysis for the outcome (Figure 2F). All pharmacologic treatments were associated with significantly higher ORs versus placebo when data were combined in the NMA, but only sumatriptan/naproxen sodium was statistically significant. According to the SUCRA, sumatriptan/naproxen sodium was ranked as the best treatment for pain freedom from two to 24 h.

### Safety analysis

Regarding AEs, 29 studies and 11 treatments are included in Figures 2B, 3B. Based on our NMA (Table 2), except for acetaminophen, the odds of AEs were higher with the remaining pharmacologic treatments than placebo (only sumatriptan, sumatriptan NS, and zolmitriptan showed statistical significance), particularly sumatriptan NS, which showed the largest OR (3.14, 95% CI: 1.22–8.09), followed by zolmitriptan (3.03, 95% CI: 1.72–5.35). Rizatriptan appears to have the fewest AEs of triptans, but its confidence interval encompasses the null value. Direct comparison and mixed comparisons were consistent. According to SUCRA (Supplementary Table 3), apart from the placebo, acetaminophen had the lowest probability of AEs (SUCRA score: 74.6), followed by rizatriptan (SUCRA score: 68.8) and ibuprofen (SUCRA score: 53.6). The lowest SUCRA score was sumatriptan NS. Most results for sensitivity analyses were generally consistent with the base case analysis.

**TABLE 2:** League table of adverse events.

Acetaminophen	​	​	0.71 (0.10, 4.85)	​	​	​	​	​	​	​	0.77 (0.11, 5.24)
0.52 (0.06, 4.66)	**Almotriptan**	​	​	​	​	​	​	​	​	​	1.88 (0.50, 6.99)
0.52 (0.06, 4.46)	1.00 (0.17, 5.91)	**Eletriptan**	​	​	​	​	​	​	​	​	1.88 (0.54, 6.48)
0.56 (0.10, 3.30)	1.08 (0.19, 6.14)	1.08 (0.20, 5.80)	**Ibuprofen**	​	​	​	​	​	0.72 (0.14, 3.50)	​	1.64 (0.46, 5.79)
0.41 (0.05, 3.65)	0.79 (0.13, 4.87)	0.79 (0.14, 4.61)	0.73 (0.13, 4.13)	**Naratriptan**	​	​	​	​	​	​	2.37 (0.65, 8.68)
0.71 (0.11, 4.71)	1.37 (0.32, 5.83)	1.37 (0.35, 5.45)	1.28 (0.34, 4.84)	1.74 (0.42, 7.25)	**Rizatriptan**	​	​	​	​	​	1.37 (0.72, 2.62)
0.40 (0.06, 2.59)	0.77 (0.19, 3.19)	0.77 (0.20, 2.97)	0.72 (0.20, 2.63)	0.97 (0.24, 3.96)	0.56 (0.23, 1.35)	**Sumatriptan**	​	​	​	​	**2.43 (1.35, 4.38)**
0.32 (0.05, 2.07)	0.62 (0.15, 2.55)	0.62 (0.16, 2.38)	0.58 (0.16, 2.10)	0.78 (0.19, 3.17)	0.45 (0.19, 1.06)	0.80 (0.36, 1.82)	**Sumatriptan NS**	​	​	​	**3.03 (1.70, 5.40)**
0.48 (0.06, 3.73)	0.91 (0.17, 4.85)	0.91 (0.18, 4.57)	0.85 (0.18, 4.09)	1.16 (0.22, 6.04)	0.67 (0.20, 2.27)	1.19 (0.35, 3.97)	1.47 (0.45, 4.87)	**Sumatriptan/naproxen**	​	​	2.06 (0.71, 5.97)
0.31 (0.05, 2.13)	0.60 (0.12, 2.98)	0.60 (0.13, 2.80)	0.56 (0.16, 1.96)	0.76 (0.15, 3.71)	0.44 (0.14, 1.38)	0.78 (0.26, 2.36)	0.97 (0.32, 2.92)	0.66 (0.16, 2.71)	**Zolmitriptan**	​	**3.40 (1.26, 9.16)**
0.52 (0.08, 3.54)	1.01 (0.23, 4.42)	1.01 (0.24, 4.14)	0.93 (0.24, 3.67)	1.27 (0.29, 5.50)	0.73 (0.28, 1.93)	1.30 (0.52, 3.29)	1.62 (0.65, 4.06)	1.10 (0.31, 3.94)	1.68 (0.51, 5.51)	**Zolmitriptan NS**	1.86 (0.89, 3.88)
0.98 (0.17, 5.75)	1.88 (0.52, 6.86)	1.88 (0.56, 6.35)	1.75 (0.55, 5.60)	2.38 (0.66, 8.51)	1.37 (0.72, 2.61)	**2.44 (1.36, 4.37)**	**3.03 (1.72, 5.35)**	2.06 (0.72, 5.89)	**3.14 (1.22, 8.09)**	1.87 (0.91, 3.84)	**Placebo**

Pairwise (upper-right portion) and network (lower-left portion) meta-analysis results are presented as estimated effect sizes for the adverse events. For the pairwise meta-analyses, outcomes are expressed as odds ratios (OR) with 95% confidence interval (CI) (OR, of > 1 indicated that the treatment specified in the row got more improvement than that specified in the column), 0 < OR < 1, the opposite. For the network meta-analysis, OR, of > 1 indicated that the treatment specified in the column got better improvement than that specified in the row, 0 < OR < 1, the opposite.95% CI, that did not contain one was considered to have a statistical difference. Bold results indicated statistical significance. * indicated a significant difference between direct and mixed comparisons. ^ indicated a significant difference between the basic model and sensitivity analysis. NS, nasal spray; SUS, suspension.

There were insufficient data to perform a quantitative comparison of drug-related AE. Because the included studies either did not report data in all intervention arms or had zero events. Thus, we characterized the available data only. The proportion of drug-related AE was small. For most studies, AEs were mild to moderate. Somnolence and nausea were the most commonly reported AEs for triptans. Most trials of triptans reported no serious adverse events or omitted this data. One trial of rizatriptan (Ho et al., 2012) reported two serious adverse events (*Escherichia coli* bacteremia and migraine). While another trial on zolmitriptan (Rothner et al., 2006) documented one severe adverse event (severe persistent headache). Additionally, five subjects (<1%) discontinued participation due to adverse reactions. However, none of these serious adverse events were deemed drug-related. Only one study (Visser et al., 2004) reported that one patient discontinued rizatriptan due to a serious laboratory adverse event (increased creatine phosphokinase) that was related to a strenuous athletic lifestyle. Another patient discontinued rizatriptan due to an ECG adverse event (elevated ST segment) that occurred on study day 110 (18 days after treatment of the 5th migraine attack) and was considered possibly drug-related.

The adverse reactions associated with triptan nasal sprays differ somewhat from those of oral formulations, with taste disturbance emerging as the most frequently reported adverse event. The overall incidence of adverse events increases with higher doses of triptans. When taste disturbance is excluded from the calculations, the overall incidence of adverse events in the triptan nasal spray treatment group is comparable to, or even lower than, that in the placebo group. Bad taste of the medication was the most common complaint; the taste may also cause nausea and vomiting, leading to withdrawal from the trial in a tiny number of patients. No serious adverse events related to the drug were observed. Twenty-three patients treated with sumatriptan nasal spray in Winner et al., ’s 2000 study (Winner et al., 2000) reported drug-related AE; disturbance of taste is the most common adverse reaction, and the incidence of all drug-related adverse reactions is positively correlated with the dosage administered. Derosier et al., ’s 2012 study (Derosier et al., 2012)reported that the only drug-related AE with an incidence of 2% was muscle tightness in 3 (2%) subjects with 85/500 mg, likely representing “triptan sensations”, and in events emerging within 72 h after dosing, a dose response was seen.

The adverse reactions reported with ibuprofen use are primarily gastrointestinal, but they are mild in severity, and there was no significant difference in adverse events observed. Reported side effects of sumatriptan-naproxen sodium include dizziness, abnormal sensations, drowsiness, nausea, dry mouth, and chest discomfort. There was one treatment-emergent serious adverse event that occurred (Lipton et al., 2009): increased headache pain that required hospitalization. This serious adverse event did not happen within 72 h of study drug dosing and was deemed by the investigator not to be drug-related.

### Additional analyses

We constructed separate networks for pediatric and adolescent migraine patients to obtain extra findings. The subgroup analysis for childhood migraine included six studies covering six treatments, showing that no acute medications, except sumatriptan nasal spray, were associated with superior efficacy to placebo in pain freedom at 2 h (Supplementary Table 12 in the Supplementary Material). The adolescent subgroup comprised 17 studies with nine interventions. Compared with placebo, sumatriptan/naproxen sodium (2.91, 95% CI: 1.87–4.53), zolmitriptan NS (2.35, 95% CI: 1.67–3.31), sumatriptan NS (1.61, 95% CI: 1.24–2.08) and rizatriptan (1.43, 95% CI: 1.13–1.81) exhibited a significant effect size on pain freedom at 2 h (Supplementary Table 13 in the Supplementary Material). When considering only oral medications, sumatriptan/naproxen sodium, ibuprofen, and rizatriptan demonstrated higher effect sizes compared to placebo, with 2.91, 2.88, and 1.51 respectively. Notably, the limited number of studies for each outcome in this analysis prevents a conclusive judgment on the effectiveness of the interventions.

### Publication bias and inconsistency

In the risk of bias assessment, 26.6% (8/30 studies), 50.0% (15/30 studies), and 23.4% (7/30 studies) had an overall low risk of bias, some concerns, and high risk of bias, respectively (Supplementary Figures 1, 2). The randomization process and measurement of the outcome led to high and unclear risks of bias, respectively. The funnel plot of publication bias across the included studies showed no obvious asymmetry (Supplementary Figure 3). There was no significant global inconsistency in the design-by-treatment model (Supplementary Table 15). For local inconsistency (Supplementary Table 16), side-splitting inconsistencies were also exhibited, but were few overall (Supplementary Table 17). The GRADE results assessment is presented in Supplementary Appendix 3. Overall, the quality of evidence for most comparisons in the current NMA ranged from low to very low.

## Discussion

Due to the specific characteristics of the pediatric and adolescent population, few RCTs have directly compared active drugs focused on this group. The majority of evidence of medicines in children comes from studies in adults (Klassen et al., 2008). We conducted an NMA, combining direct and indirect comparisons to yield more accurate comparisons than RCTs and traditional meta-analyses. We hope this study can provide valuable references for the clinical pharmacological management of migraine in children and adolescents.

Pain freedom at 2 h stands as a pivotal metric in evaluating the efficacy of acute migraine treatment. Our findings show that sumatriptan/naproxen sodium, ibuprofen, zolmitriptan nasal spray, sumatriptan nasal spray, and rizatriptan demonstrated superiority compared with placebo. Dihydroergotamine was associated with the highest effect size and SUCRA values, but its confidence interval included null values. For pain freedom from two to 24 h, only sumatriptan/naproxen sodium showed a significant effect size compared with placebo. Ibuprofen exhibited the highest effect size for pain relief at 2 hours. None of the included drugs were found to reduce the use of rescue drugs from 2 to 24 h. Regarding safety and tolerability, the lowest OR indicates a more favorable risk profile compared to the estimate of acetaminophen for other investigated interventions. Zolmitriptan was associated with the highest risk of AEs among all treatments.

Non-steroidal anti-inflammatory drugs are typically employed as the initial pharmacological treatment for acute migraine in children (Ho et al., 2012). Paracetamol (acetaminophen) and ibuprofen are common over-the-counter medications for pediatric migraine, having demonstrated efficacy and safety in the acute treatment of children under 12 years of age (Brenner and Lewis, 2008; Kacperski et al., 2016). The American Academy of Neurology (AAN) guidelines recommend ibuprofen (10 mg/kg) as an initial treatment option (Level B) (Oskoui et al., 2019), but do not formally recommend acetaminophen, likely due to the scarcity of well-designed controlled trials of acetaminophen in this population. Our NMA revealed that ibuprofen showed favorable estimates versus placebo, with a favorable efficacy/tolerability balance. The advantage of acetaminophen lies in the low risk of AEs. In clinical practice, acetaminophen is a potential alternative, particularly for patients with contraindications to NSAIDs. Further trials in this population would help support its use as a treatment option (Jeric et al., 2018).

Triptans are highly selective 5-hydroxytryptamine (5-HT) receptor agonists that exert their effects primarily through the 5-HT1B/1D receptors, likely by preventing activation and/or sensitization of central second-order neurons in the trigeminal nucleus caudalis and reducing the release of pro-inflammatory mediators at peripheral trigeminal nerve endings (Levy et al., 2004; Chatterjee and Blume, 2024). The AAN, European Academy of Neurology (EAN), and International Headache Society (IHS) recommend zolmitriptan and sumatriptan NS for the treatment of migraine in adolescents (Hassan et al., 2026; Oskoui et al., 2019), as these are the longer-established agents in this population. In the current study, both oral and nasal spray formulations of zolmitriptan and sumatriptan demonstrated higher effect sizes compared to placebo (Iannone et al., 2022). Sumatriptan nasal spray was also the only drug to show significant efficacy compared to placebo in the children’s subgroup. Nasal spray formulations are particularly suitable for patients experiencing significant gastroparesis during headache episodes. Rizatriptan and almotriptan should be considered more cautiously. In this NMA, rizatriptan was more effective than placebo in pain freedom at 2 hours after administration, whereas almotriptan did not show a significant improvement compared with placebo. The inconsistent acceptance may be related to the lack of robust, large-scale RCTs in this age group. Further high-quality pediatric trials will help determine the efficacy and safety of rizatriptan and almotriptan in this age group.

Sumatriptan/naproxen sodium is a combination drug consisting of sumatriptan and naproxen (an NSAID). Multiple RCTs in adults have confirmed that sumatriptan/naproxen sodium effectively alleviates migraine symptoms (Winner et al., 2015; Brandes et al., 2007; Calhoun and Ford, 2014). In 2008, sumatriptan/naproxen sodium was approved for marketing by the FDA and is now approved in several countries (Lipton et al., 2009). The present NMA showed that sumatriptan/naproxen sodium is more likely than placebo to result in headache pain-free status at 2 h. When a single treatment was insufficient, triptans were often recommended for use combined with NSAIDs, based on existing evidence suggesting a potential synergistic effect (Loder and Weizenbaum, 2014). For adolescent patients who respond inadequately to NSAIDs, sumatriptan/naproxen sodium should be considered.

Dihydroergotamine is commonly used for the acute treatment of migraine and other headaches in adults. Evidence supports dihydroergotamine for acute and refractory headaches in children, but lacks high-quality RCT results (Linder, 1994; Srouji et al., 2021). Although ergometrine derivatives have relatively poor tolerability, they have a rapid onset of action, sustained effect, and low risk of medication-overuse headache, demonstrating good clinical potential (Silberstein et al., 2020). For refractory and chronic migraine in children, intravenous administration of dihydroergotamine can provide some improvement.

The most recently marketed drugs, such as lasmiditan and small-molecule calcitonin gene-related peptide receptor antagonists (rimegepant, ubrogepant, atogepant, and zavegepant), are emerging and recently approved acute antimigraine therapies. These therapies are now undergoing evaluation in pediatric clinical trials (Moreno-Ajona et al., 2021; Wells-Gatnik et al., 2024); however, their safety and efficacy in the pediatric population remain formally unestablished pending the release of definitive trial data.

### Strengths and limitations

Given certain limitations, our findings should be considered with caution. First, the large number of trials with moderate-to-high risk of bias undoubtedly diminished the credibility of the findings. This resulted in the most significant comparisons in subsequent CINeMA evaluations, yielding conclusions of low or very low confidence. Second, we included a small number of studies and sample sizes for some interventions, which may lead to instability, especially when using random effects models. The inclusion of crossover trials introduces potential methodological heterogeneity. These trials may provide more conservative estimates of treatment efficacy due to possible carry-over effects, which can dilute the contrast between interventions. Another significant limitation is the heterogeneity of the patient population. Since the majority of included trials did not perform age stratification, we were unable to conduct a three-arm independent analysis (children, adolescents, and combined groups). Due to variations in study design, age groups, and dosing strategies, the transitivity assumption of the NMA may not have been fully satisfied. Consequently, the synthesized evidence presented herein predominantly reflects the efficacy profile in adolescents. Caution is strongly advised when extrapolating these findings to younger children (<12 years), as pharmacokinetics, pharmacodynamics, and placebo responses may differ substantially in this age group. In addition, we were unable to perform Egger’s test due to limited studies per pairwise comparison, which restricts formal quantification of publication bias. Therefore, we relied on visual inspection of comparison-adjusted funnel plots, and readers should be aware that small-study effects cannot be entirely excluded.

## Conclusion

This NMA evaluated the relative efficacy and safety of drugs for the acute treatment of migraine in children and adolescents. Ibuprofen can effectively relieve symptoms, characterized by a favorable benefit-risk profile. Sumatriptan and zolmitriptan nasal sprays also exhibited robust efficacy, specifically among populations with prominent nausea and vomiting. Sumatriptan/naproxen sodium merits consideration, especially in patients exhibiting an inadequate response to monotherapy. Dihydroergotamine demonstrated potential benefits in refractory and chronic migraine; however, high-quality studies are warranted to validate these findings.

## Data Availability

The original contributions presented in the study are included in the article/Supplementary Material, further inquiries can be directed to the corresponding authors.
