# Supplementary material for: Drug interventions for acute treatment of pediatric migraine: a systematic review and network meta-analysis
Source: Front Pharmacol. 2026 Jul 15;17:1851994. doi: 10.3389/fphar.2026.1851994 (PMC13416359; doi:10.3389/fphar.2026.1851994)
Supplement: Supplementary file 1 [file Supplementaryfile1.docx]

# **Supplementary material**

eAppendix 1. PRISMA NMA checklist

eAppendix 2. Search strategies

eAppendix 3. GRADE ratings for primary network

eTable 1. Baseline demographics characteristics

eTable 2 - 6. SUCRA of each outcome for anti-migraine-specific treatments

eTable 7 - 9. Head-to-head comparisons of secondary outcomes

eTable 10. Sensitivity analysis of pain freedom at 2 hours

eTable 11. Sensitivity analysis of adverse events

eTable 12: Subgroup analysis of pain freedom at 2 hours in Children

eTable 13: Subgroup analysis of pain freedom at 2 hours in Adolescents

eTable 14: Subgroup analysis of oral pharmacological agents

eTable 15: Design-by-treatment interaction model for inconsistency of network meta-analysis

eTable 16. Significant loop-specific inconsistencies of network meta-analysis

eTable 17. Significant side-splitting inconsistencies of network meta-analysis

eFigure 1. Overview of risk of bias

eFigure 2. Detailed risk of bias in each study

eFigure 3. Funnel plot of changes in each outcome

eReferences

**eAppendix 1:** PRISMA NMA checklist

| **Section/Topic** | **Item #** | **Checklist Item** | **Reported on Page #** |
| --- | --- | --- | --- |
| **TITLE** |  |  |  |
| Title | 1 | Identify the report as a systematic review *incorporating a network meta-analysis (or related form of meta-analysis).* | 1 |
|  |  |  |  |
| **ABSTRACT** |  |  |  |
| Structured summary | 2 | Provide a structured summary including, as applicable:  **Background:** main objectives  **Methods:** data sources; study eligibility criteria, participants, and interventions; study appraisal; and *synthesis methods, such as network meta-analysis.*  **Results:** number of studies and participants identified; summary estimates with corresponding confidence/confidence intervals; *treatment rankings may also be discussed. Authors may choose to summarize pairwise comparisons against a chosen treatment included in their analyses for brevity.*  **Discussion/Conclusions:** limitations; conclusions and implications of findings.  **Other:** primary source of funding; systematic review registration number with registry name. | 1 |
|  |  |  |  |
| **INTRODUCTION** |  |  |  |
| Rationale | 3 | Describe the rationale for the review in the context of what is already known*, including mention of why a network meta-analysis has been conducted.* | 2 |
| Objectives | 4 | Provide an explicit statement of questions being addressed, with reference to participants, interventions, comparisons, outcomes, and study design (PICOS). | 4 |
|  |  |  |  |
| **METHODS** |  |  |  |
| Protocol and registration | 5 | Indicate whether a review protocol exists and if and where it can be accessed (e.g., Web address); and, if available, provide registration information, including registration number. | 3 |
| Eligibility criteria | 6 | Specify study characteristics (e.g., PICOS, length of follow-up) and report characteristics (e.g., years considered, language, publication status) used as criteria for eligibility, giving rationale. *Clearly describe eligible treatments included in the treatment network, and note whether any have been clustered or merged into the same node (with justification).* | 4 |
| Information sources | 7 | Describe all information sources (e.g., databases with dates of coverage, contact with study authors to identify additional studies) in the search and date last searched. | 4 |
| Search | 8 | Present full electronic search strategy for at least one database, including any limits used, such that it could be repeated. | 4 |
| Study selection | 9 | State the process for selecting studies (i.e., screening, eligibility, included in systematic review, and, if applicable, included in the meta-analysis). | 4 |
| Data collection process | 10 | Describe method of data extraction from reports (e.g., piloted forms, independently, in duplicate) and any processes for obtaining and confirming data from investigators. | 4 |
| Data items | 11 | List and define all variables for which data were sought (e.g., PICOS, funding sources) and any assumptions and simplifications made. | 4 |
| **Geometry of the network** | **S1** | Describe methods used to explore the geometry of the treatment network under study and potential biases related to it. This should include how the evidence base has been graphically summarized for presentation, and what characteristics were compiled and used to describe the evidence base to readers. | 5-6 |
| Risk of bias within individual studies | 12 | Describe methods used for assessing risk of bias of individual studies (including specification of whether this was done at the study or outcome level), and how this information is to be used in any data synthesis. | 5 |
| Summary measures | 13 | State the principal summary measures (e.g., risk ratio, difference in means). *Also describe the use of additional summary measures assessed, such as treatment rankings and surface under the cumulative ranking curve (SUCRA) values, as well as modified approaches used to present summary findings from meta-analyses.* | 5-6 |
| Planned methods of analysis | 14 | Describe the methods of handling data and combining results of studies for each network meta-analysis. This should include, but not be limited to:   - *Handling of multi-arm trials;* - *Selection of variance structure;* - *Selection of prior distributions in Bayesian analyses; and* - *Assessment of model fit.* | 5-6 |
| **Assessment of Inconsistency** | **S2** | Describe the statistical methods used to evaluate the agreement of direct and indirect evidence in the treatment network(s) studied. Describe efforts taken to address its presence when found. | 6 |
| Risk of bias across studies | 15 | Specify any assessment of risk of bias that may affect the cumulative evidence (e.g., publication bias, selective reporting within studies). | 12 |
| Additional analyses | 16 | Describe methods of additional analyses if done, indicating which were pre-specified. This may include, but not be limited to, the following:   - Sensitivity or subgroup analyses; - Meta-regression analyses; - *Alternative formulations of the treatment network; and* - *Use of alternative prior distributions for Bayesian analyses (if applicable).* | 9 |
| **RESULTS†** |  |  |  |
| Study selection | 17 | Give numbers of studies screened, assessed for eligibility, and included in the review, with reasons for exclusions at each stage, ideally with a flow diagram. | Not applicable |
| **Presentation of network structure** | **S3** | Provide a network graph of the included studies to enable visualization of the geometry of the treatment network. | Figure 2 |
| **Summary of network geometry** | **S4** | Provide a brief overview of characteristics of the treatment network. This may include commentary on the abundance of trials and randomized patients for the different interventions and pairwise comparisons in the network, gaps of evidence in the treatment network, and potential biases reflected by the network structure. | 7-8, eTable 1 |
| Study characteristics | 18 | For each study, present characteristics for which data were extracted (e.g., study size, PICOS, follow-up period) and provide the citations. | Not applicable |
| Risk of bias within studies | 19 | Present data on risk of bias of each study and, if available, any outcome level assessment. | 9, eTable 15-17,eFigure 1-3 |
| Results of individual studies | 20 | For all outcomes considered (benefits or harms), present, for each study: 1) simple summary data for each intervention group, and 2) effect estimates and confidence intervals. *Modified approaches may be needed to deal with information from larger networks.* | 6-8, Figure 3 |
| Synthesis of results | 21 | Present results of each meta-analysis done, including confidence/confidence intervals. *In larger networks, authors may focus on comparisons versus a particular comparator (e.g. placebo or standard care), with full findings presented in an appendix. League tables and forest plots may be considered to summarize pairwise comparisons.* If additional summary measures were explored (such as treatment rankings), these should also be presented. | 6-8, Table1-2, Figure 2, eTable 7-9 |
| **Exploration for inconsistency** | **S5** | Describe results from investigations of inconsistency. This may include such information as measures of model fit to compare consistency and inconsistency models, *P* values from statistical tests, or summary of inconsistency estimates from different parts of the treatment network. | eTable 15-17 |
| Risk of bias across studies | 22 | Present results of any assessment of risk of bias across studies for the evidence base being studied. | eFigure 1-2 |
| Results of additional analyses | 23 | Give results of additional analyses, if done (e.g., sensitivity or subgroup analyses, meta-regression analyses*, alternative network geometries studied, alternative choice of prior distributions for Bayesian analyses,* and so forth). | 9, eTable 12-14 |
|  |  |  |  |
| **DISCUSSION** |  |  |  |
| Summary of evidence | 24 | Summarize the main findings, including the strength of evidence for each main outcome; consider their relevance to key groups (e.g., healthcare providers, users, and policy-makers). | 10 |
| Limitations | 25 | Discuss limitations at study and outcome level (e.g., risk of bias), and at review level (e.g., incomplete retrieval of identified research, reporting bias). *Comment on the validity of the assumptions, such as transitivity and consistency. Comment on any concerns regarding network geometry (e.g., avoidance of certain comparisons).* | 12 |
| Conclusions | 26 | Provide a general interpretation of the results in the context of other evidence, and implications for future research. | 12 |
|  |  |  |  |
| **FUNDING** |  |  |  |
| Funding | 27 | Describe sources of funding for the systematic review and other support (e.g., supply of data); role of funders for the systematic review. This should also include information regarding whether funding has been received from manufacturers of treatments in the network and/or whether some of the authors are content experts with professional conflicts of interest that could affect use of treatments in the network. | 13 |

## eAppendix 2: Search strategies

**PubMed from inception until 1 August April 2025**

| **#** | **Searches** | **Results** |
| --- | --- | --- |
| 1 | Migraine Disorders[Mesh] | 34,349 |
| 2 | Migraine disorders[Title/Abstract] OR Disorder, Migraine[Title/Abstract] OR Disorders, Migraine[Title/Abstract] OR Migraine Disorder[Title/Abstract] OR Migraine[Title/Abstract] OR Migraines[Title/Abstract] OR Migraine Headache[Title/Abstract] OR Headache, Migraine[Title/Abstract] OR Headaches, Migraine[Title/Abstract] OR Migraine Headaches[Title/Abstract] OR Acute Confusional Migraine[Title/Abstract] OR Acute Confusional Migraines[Title/Abstract] OR Migraine, Acute Confusional[Title/Abstract] OR Migraines, Acute Confusional[Title/Abstract] OR Status Migrainosus[Title/Abstract] OR Hemicrania Migraine[Title/Abstract] OR Hemicrania Migraines[Title/Abstract] OR Migraine, Hemicrania[Title/Abstract] OR Migraines, Hemicrania[Title/Abstract] OR Migraine Variant[Title/Abstract] OR Migraine Variants[Title/Abstract] OR Variant, Migraine[Title/Abstract] OR Variants, Migraine[Title/Abstract] OR Sick Headache[Title/Abstract] OR Headache, Sick[Title/Abstract] OR Headaches, Sick[Title/Abstract] OR Sick Headaches[Title/Abstract] OR Abdominal Migraine[Title/Abstract] OR Abdominal Migraines[Title/Abstract] OR Migraine, Abdominal[Title/Abstract] OR Migraines, Abdominal[Title/Abstract] OR Cervical Migraine Syndrome[Title/Abstract] OR Cervical Migraine Syndromes[Title/Abstract] OR Migraine Syndrome, Cervical[Title/Abstract] OR Migraine Syndromes, Cervical[Title/Abstract] | 48,071 |
| 3 | 1 OR 2 | 52,295 |
| 4 | child* OR pediatric* OR paediatric* OR infant* OR juvenile* adolescen* OR developmental age | 4,526,963 |
| 5 | (clinical[tiab] AND trial[tiab]) OR clinical trials as topic[mesh] OR clinical trial[pt] OR random*[tiab] OR random allocation[mesh] OR therapeutic use[sh] | 6,874,167 |
| 6 | animals[Mesh] NOT humans[Mesh] | 5,368,421 |
| 7 | 5 NOT 6 | 6,049,291 |
| 8 | 3 AND 4 AND7 | 2,361 |

**Embase from inception until 1 August 2025**

| **#** | **Searches** | **Results** |
| --- | --- | --- |
| 1 | 'migraine disorders'/exp | 91,671 |
| 2 | 'migraine disorders':ab,ti,kw OR 'disorder, migraine':ab,ti,kw OR 'disorders, migraine':ab,ti,kw OR 'migraine disorder':ab,ti,kw OR 'migraine':ab,ti,kw OR 'migraines':ab,ti,kw OR 'migraine headache':ab,ti,kw OR 'headache, migraine':ab,ti,kw OR 'headaches, migraine':ab,ti,kw OR 'migraine headaches':ab,ti,kw OR 'acute confusional migraine':ab,ti,kw OR 'acute confusional migraines':ab,ti,kw OR 'migraine, acute confusional':ab,ti,kw OR 'migraines, acute confusional':ab,ti,kw OR 'status migrainosus':ab,ti,kw OR 'hemicrania migraine':ab,ti,kw OR 'hemicrania migraines':ab,ti,kw OR 'migraine, hemicrania':ab,ti,kw OR 'migraines, hemicrania':ab,ti,kw OR 'migraine variant':ab,ti,kw OR 'migraine variants':ab,ti,kw OR 'variant, migraine':ab,ti,kw OR 'variants, migraine':ab,ti,kw OR 'sick headache':ab,ti,kw OR 'headache, sick':ab,ti,kw OR 'headaches, sick':ab,ti,kw OR 'sick headaches':ab,ti,kw OR 'abdominal migraine':ab,ti,kw OR 'abdominal migraines':ab,ti,kw OR 'migraine, abdominal':ab,ti,kw OR 'migraines, abdominal':ab,ti,kw OR 'cervical migraine syndrome':ab,ti,kw OR 'cervical migraine syndromes':ab,ti,kw OR 'migraine syndrome, cervical':ab,ti,kw OR 'migraine syndromes, cervical':ab,ti,kw | 77,480 |
| 3 | 1 OR 2 | 100,666 |
| 4 | child* OR pediatric* OR paediatric* OR infant* OR juvenile* adolescen* OR developmental age | 719,540 |
| 5 | 'clinical':ti,ab AND 'trial':ti,ab OR 'clinical trial'/exp OR random* OR 'drug therapy':lnk | 8,298,490 |
| 12 | 3 AND 4 AND 5 | 1,146 |

**Cochrane Library from inception until 1 AugustApril 2025**

| **#** | **Searches** | **Results** |
| --- | --- | --- |
| 1 | MeSH descriptor: [Migraine disorders] explode all trees | 3,932 |
| 2 | migraine disorders OR disorder, migraine OR disorders, migraine OR migraine disorder OR migraine OR migraines OR migraine headache OR headache, migraine OR headaches, migraine OR migraine headaches OR acute confusional migraine OR acute confusional migraines OR migraine, acute confusional OR migraines, acute confusional OR status migrainosus OR hemicrania migraine OR hemicrania migraines OR migraine, hemicrania OR migraines, hemicrania OR migraine variant OR migraine variants OR variant, migraine OR variants, migraine OR sick headache OR headache, sick OR headaches, sick OR sick headaches OR abdominal migraine OR abdominal migraines OR migraine, abdominal OR migraines, abdominal OR cervical migraine syndrome OR cervical migraine syndromes OR migraine syndrome, cervical OR migraine syndromes, cervical | 10,892 |
| 3 | 1 OR 2 | 10,892 |
| 4 | child* OR pediatric* OR paediatric* OR infant* OR juvenile* adolescen* OR developmental age | 288,254 |
| 5 | 3 AND 4 | 1,159 |
| 6 | In trials | 853 |

eAppendix 3: GRADE ratings for Primary Outcome Network

The Grading of Recommendations Assessment, Development, and Evaluation (GRADE) methods provide confidence assessments of the results of systematic reviews and meta-analyses and have been widely accepted and applied [1, 2]. However, the complexity of the methods and the lack of suitable software have limited their adoption. Confidence in Network Meta-Analysis (CINeMA) [3] is an approach for determining confidence in the results of an NMA broadly based on GRADE, with several conceptual and semantic differences. It covers 6 domains: (A) within-study bias, (B) reporting bias, (C) indirectness, (D) imprecision, (E) heterogeneity, and (F) incoherence. Only the reviewer needs to input the within-study bias and indirectness at the study level. The three levels (no concerns, some concerns, or major concerns) for each domain can be judged according to user-defined rules. The judgments for each domain are eventually summarized to obtain 4 levels of confidence for each pair of comparisons (very low, low, moderate, or high). we examined the certainty of the evidence for the network estimate in line with the following criteria:

**Within-study bias**: CINeMA combines the studies’ contributions with the risk of bias judgments to evaluate within-study bias for each estimate from an NMA. It uses the percentage contribution matrix to approximate the contribution of each study. Then it computes the percentage contribution from studies judged to be at low, moderate, and high risk of bias. We categorized the overall risk of bias in each study. Then based on the results of the Cochrane Risk of Bias version 2 (RoB2) for randomized trials [4], we allocate the values of “low,” “some concern,” and “high,” as 1, 2, and 3, respectively, and input into the CINeMA web application [5].

**Reporting bias**: Although the CINeMA approach suggested some conditions associated with suspected reporting bias, this is still highly subjective. Therefore, by referring to previous NMA studies [6, 7], a comparison-adjusted funnel plot with an accompanying Egger test for asymmetry was calculated.

**Indirectness**: We judged that there was no concern in this domain as the included studies matched our inclusion criteria and study questions.

**Imprecision**: CINeMA compares the treatment effects included in the 95% confidence interval with the range of equivalence. Due to the absence of previous analyses that could be referenced, we considered a clinically meaningful threshold for odds ratios (OR) to be 0.

**Heterogeneity**: As for imprecision, the CINeMA approach to heterogeneity involves comparisons of results with the pre-specified range of clinical equivalence.

**Incoherence**: As for heterogeneity, the CINeMA approach to incoherence considers the impact on clinical implications based on visual inspection of the 95% confidence intervals of direct and indirect ORs and the range of equivalence.

**Summarizing judgments across the 6 domains**: For each pair comparison, one may start at high confidence and drop the confidence level by 1 step for each domain with some concerns, and by 2 levels for each domain with major concerns. However, it is essential to note that domains are interconnected. The 6 CINeMA domains should therefore be considered jointly rather than in isolation, avoiding downgrading the overall level of confidence more than once for related concerns. Therefore, in the GRADE of the current NMA, for the first three domains, the downgrading was no more than two levels; the same was performed for the last three domains.

**References:**

1. Guyatt, G., A. D. Oxman, E. A. Akl, R. Kunz, G. Vist, J. Brozek, S. Norris, Y. Falck-Ytter, P. Glasziou, H. Debeer, R. Jaeschke, D. Rind, J. Meerpohl, P. Dahm, and H. J. Schunemann (2011) GRADE guidelines: 1. Introduction-GRADE evidence profiles and summary of findings tables. J Clin Epidemiol. 64(4): 383-94.

2. Guyatt, G. H., A. D. Oxman, G. E. Vist, R. Kunz, Y. Falck-Ytter, P. Alonso-Coello, and H. J. Schunemann (2008) GRADE: an emerging consensus on rating quality of evidence and strength of recommendations. BMJ. 336(7650): 924-6.

3. Nikolakopoulou, A., Jpt Higgins, T. Papakonstantinou, A. Chaimani, Giovane C. Del, M. Egger, and G. Salanti (2020) CINeMA: An approach for assessing confidence in the results of a network meta-analysis. PLoS Med. 17(4): e1003082.

4. Sterne, Jac, J. Savovic, M. J. Page, R. G. Elbers, N. S. Blencowe, I. Boutron, C. J. Cates, H. Y. Cheng, M. S. Corbett, S. M. Eldridge, J. R. Emberson, M. A. Hernan, S. Hopewell, A. Hrobjartsson, D. R. Junqueira, P. Juni, J. J. Kirkham, T. Lasserson, T. Li, A. Mcaleenan, B. C. Reeves, S. Shepperd, I. Shrier, L. A. Stewart, K. Tilling, I. R. White, P. F. Whiting, and Jpt Higgins (2019) RoB 2: a revised tool for assessing the risk of bias in randomised trials. BMJ. 366: l4898.

5. Papakonstantinou, T., A. Nikolakopoulou, Jpt Higgins, M. Egger, and G. Salanti (2020) CINeMA: Software for semiautomated assessment of the confidence in the results of network meta-analysis. Campbell Syst Rev. 16(1): e1080.

6. Locher, C., J. Kossowsky, H. Koechlin, T. L. Lam, J. Barthel, C. B. Berde, J. Gaab, G. Schwarzer, K. Linde, and K. Meissner (2020) Efficacy, Safety, and Acceptability of Pharmacologic Treatments for Pediatric Migraine Prophylaxis: A Systematic Review and Network Meta-analysis. JAMA Pediatr. 174(4): 341-349.

7. Koechlin, H., J. Kossowsky, T. L. Lam, J. Barthel, J. Gaab, C. B. Berde, G. Schwarzer, K. Linde, K. Meissner, and C. Locher (2021) Nonpharmacological Interventions for Pediatric Migraine: A Network Meta-analysis. Pediatrics. 147(4).

**Pain freedom at 2 hours**


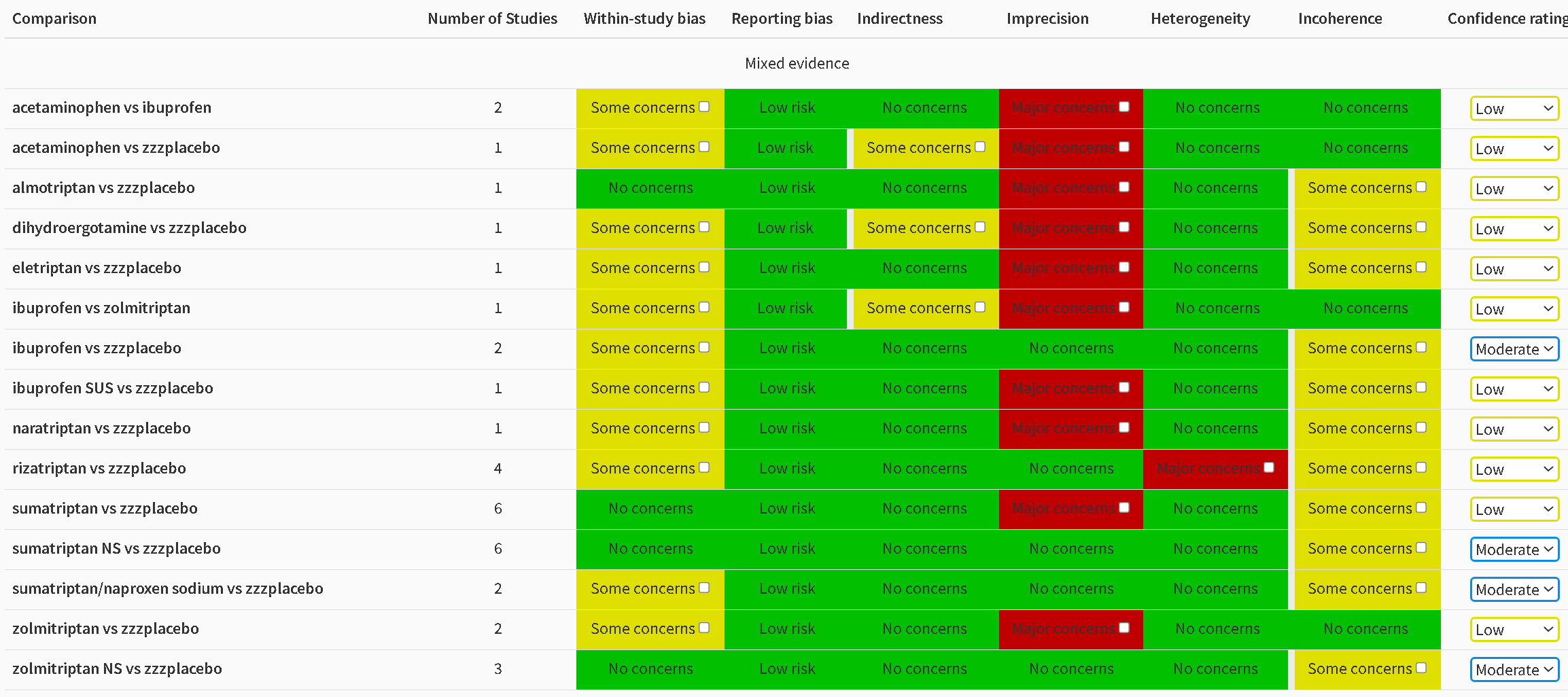


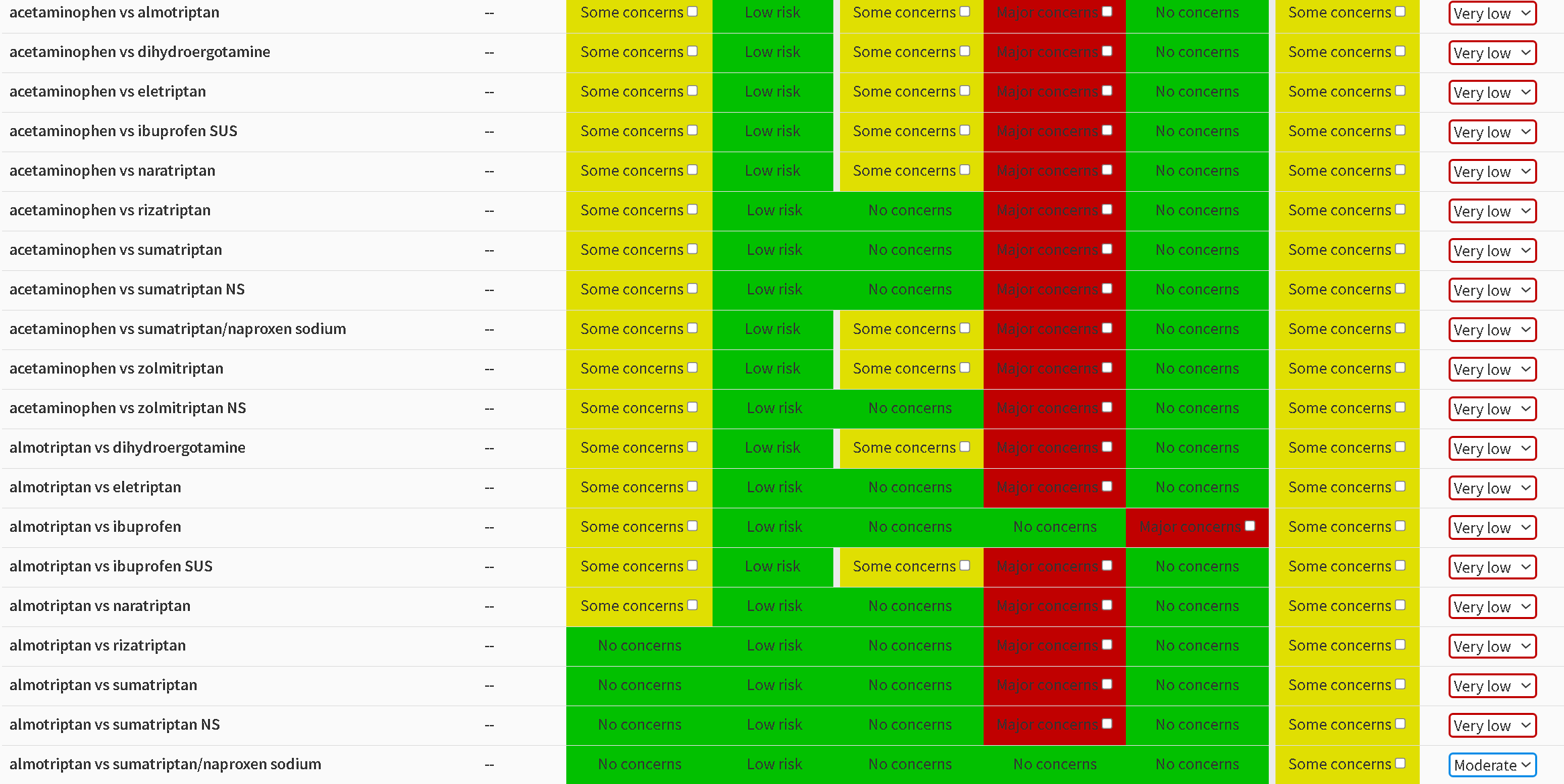


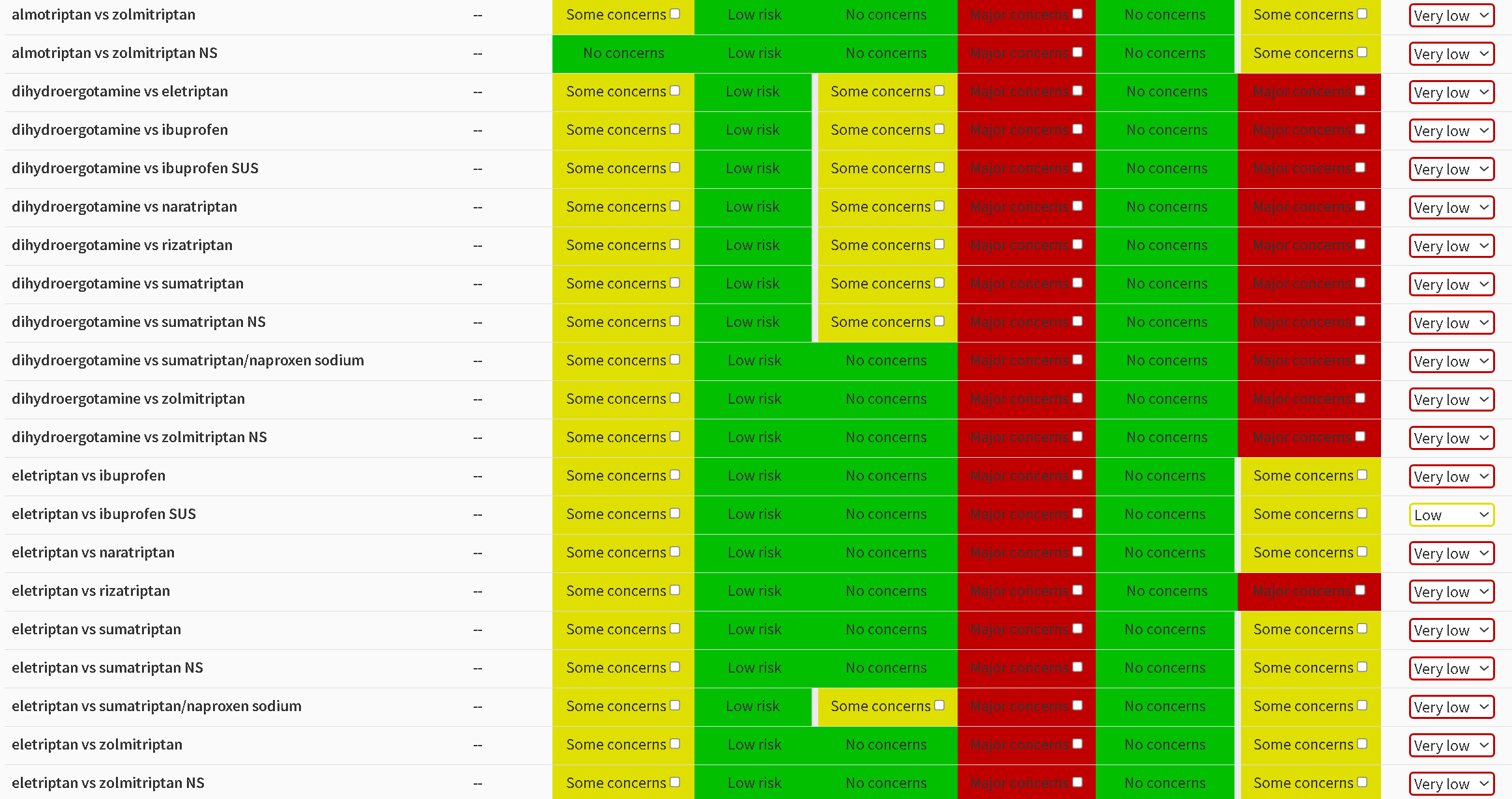


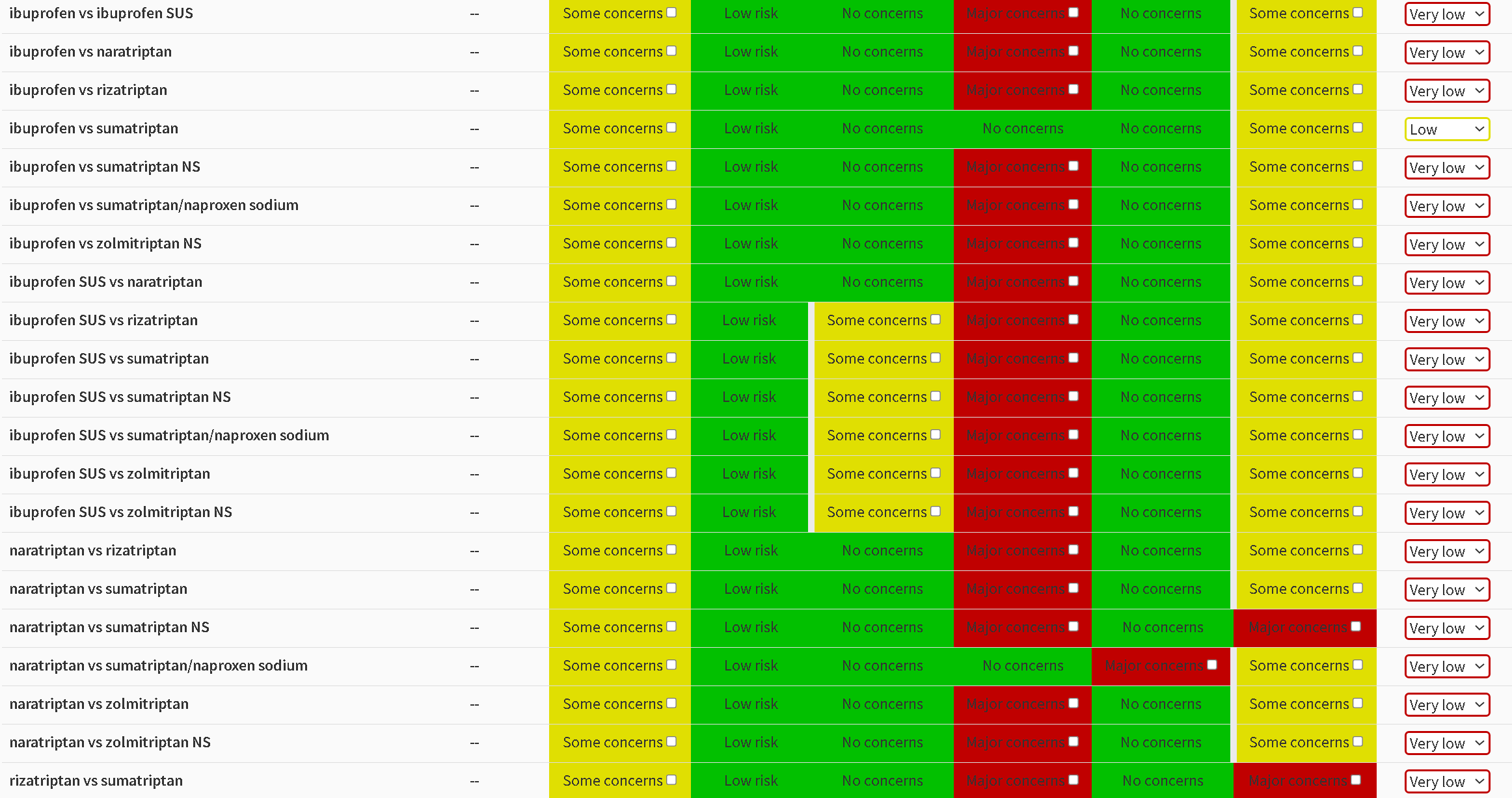


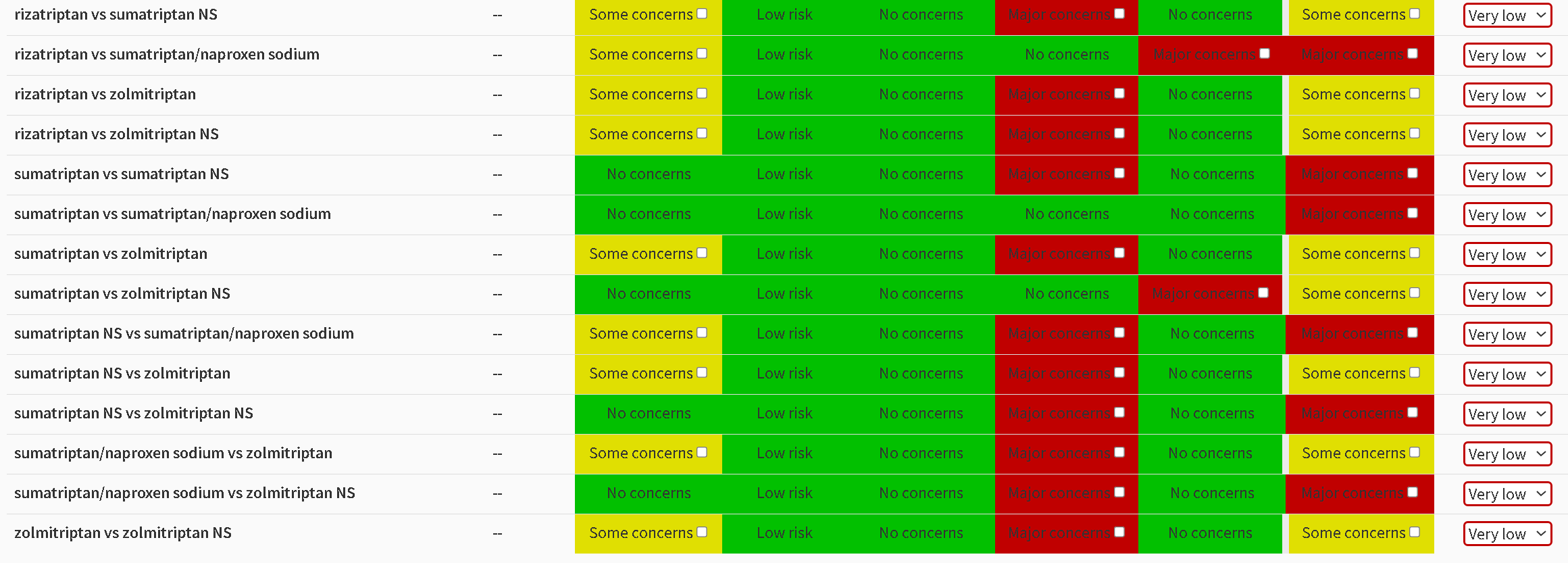


**Adverse Events**


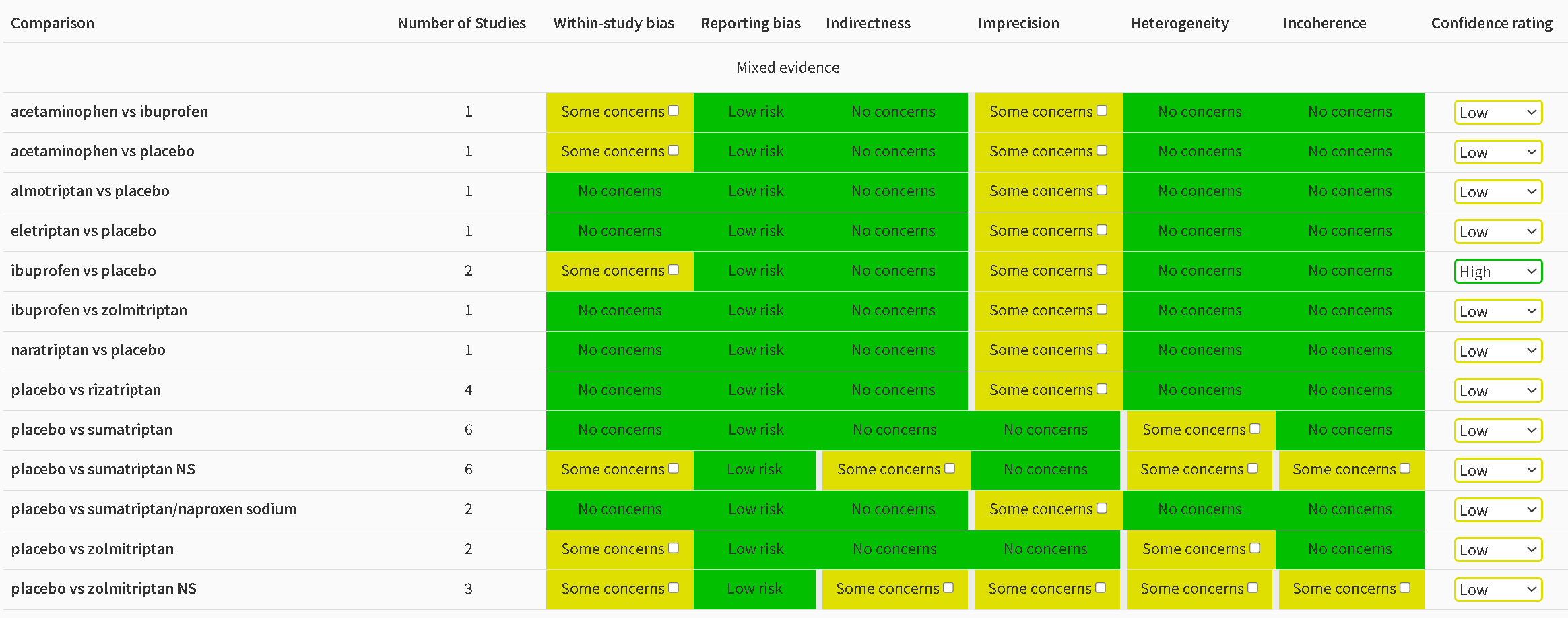


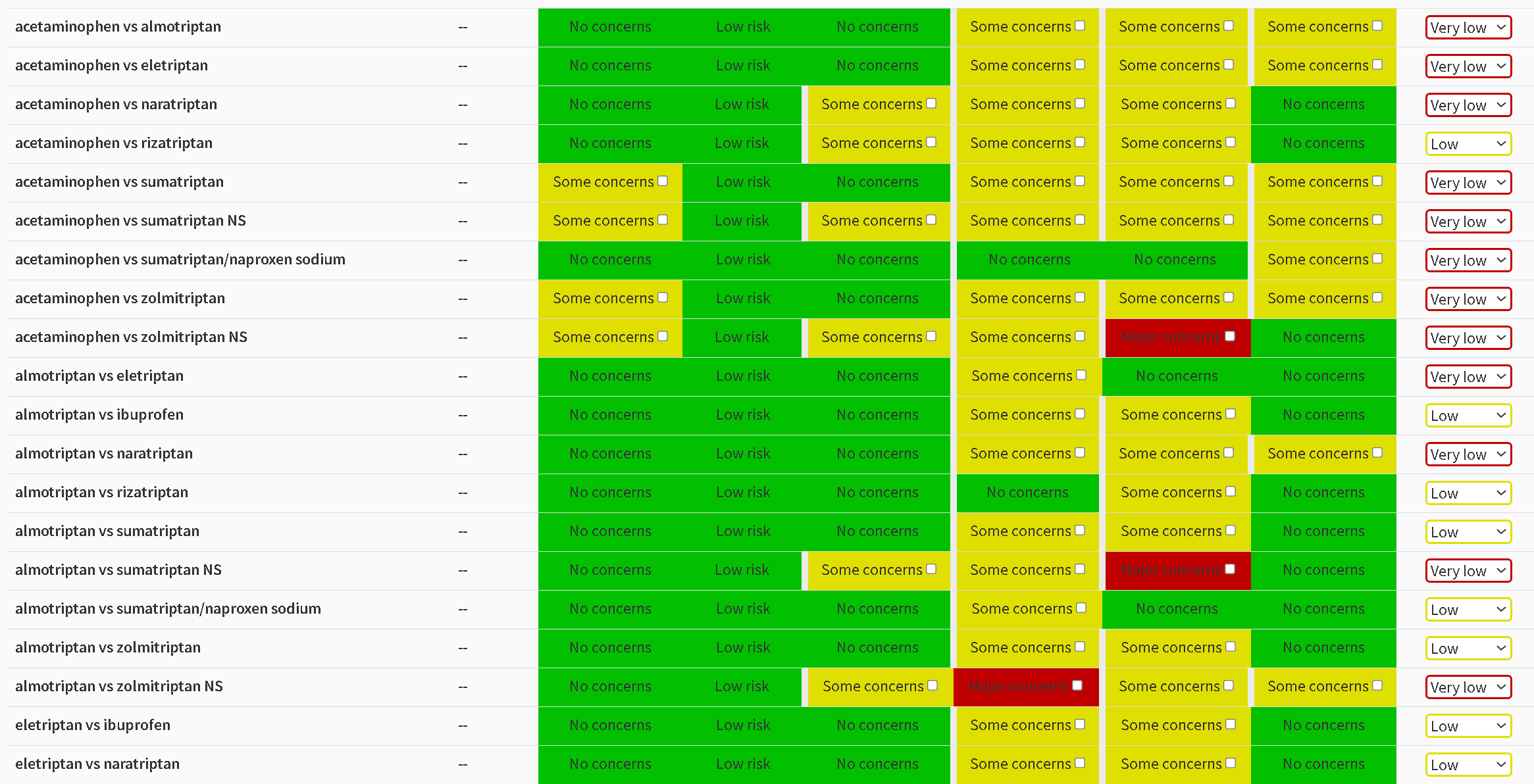


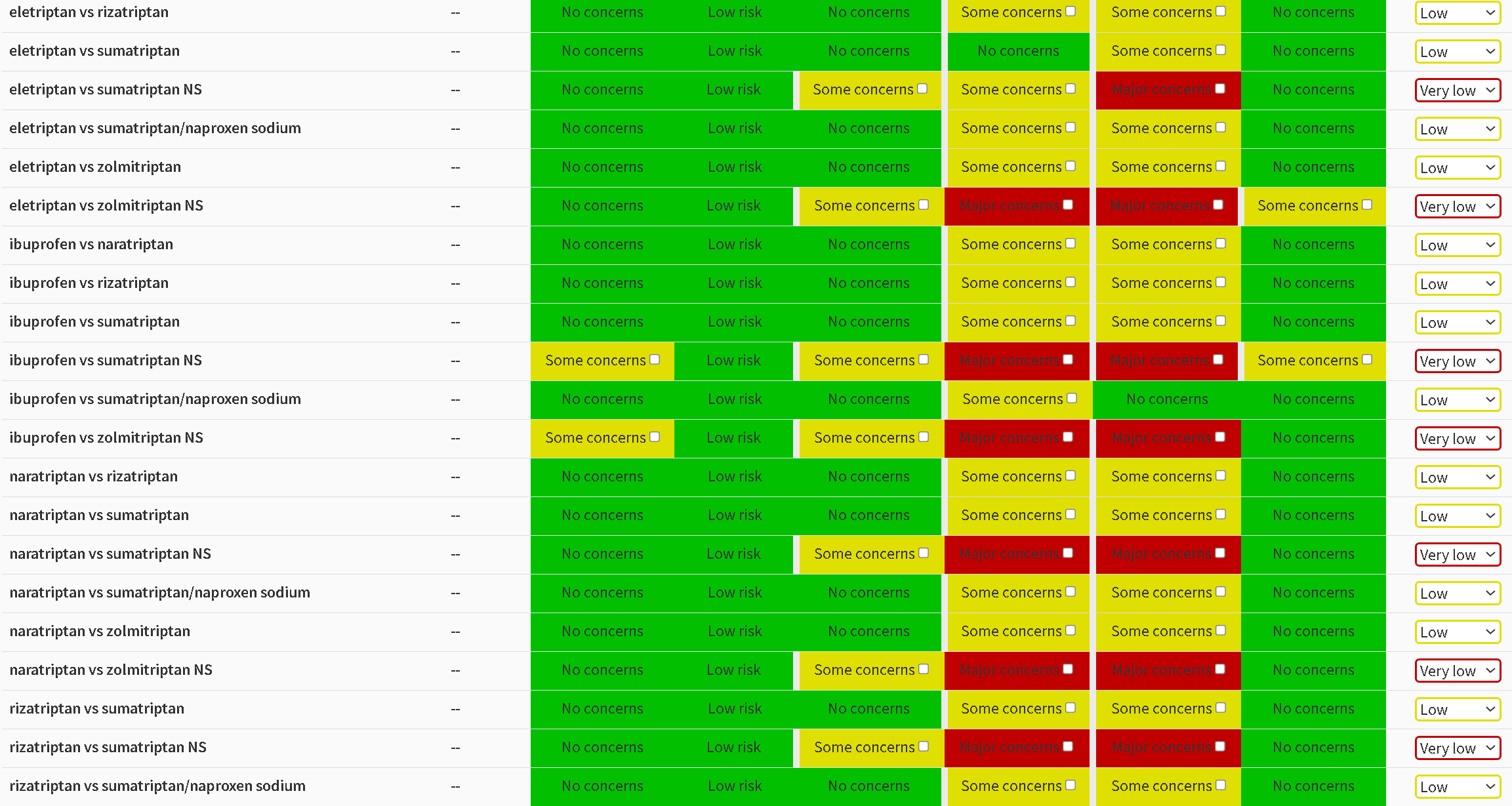


## eTable 1. Baseline demographics characteristics

| **Studies included** | **Study Design** | **Diagnostic criteria** | **Agent** | **Route** | **Intervention, Regimens, and routes** | **Sample** | **Child. (< 12 yrs)** | **Adolesc. (12‐17 yrs)** | **female, n%** | **Mean age** | **Aura, n%** | **Use of preventive medications** | **Risk of bias** |
| --- | --- | --- | --- | --- | --- | --- | --- | --- | --- | --- | --- | --- | --- |
|  |  |  |  |  |  |  |  |  |  |  |  |  |  |
|  |  |  |  |  |  |  |  |  |  |  |  |  |  |
| Ahonen 2004 [1] | Randomized, double‐blind, placebo‐controlled, two‐way cross‐over trial in Finland | IHS | Sumatriptan | IN | Sumatriptan 10 mg (weight 20 to 39 kg) or 20 mg (≥ 40 kg) versus placebo | 83 | Yes | Yes | 63% | 12.4 | 29% | NR | Low |
| Ahonen 2006 [2] | Randomized, placebo‐controlled, double‐blind, 3‐way cross‐over trial in Finland | IHS | Rizatriptan | Po | Rizatriptan 5 mg (weight 20 to 39 kg) or rizatriptan 10 mg (weight ≥ 40 kg) and placebo | 96 | Yes | Yes | 66% | 12.1 | 33% | NR | Low |
| Callenbach 2007[2] | Randomized, double‐blind, placebo‐controlled, cross‐over, 2‐attack study in the Netherlands | IHS | Sumatriptan | IN | Each participant treated 2 attacks ‐ 1 with sumatriptan (10 mg if < 40 kg or 20 mg if > 40 kg) and 1 with placebo | 46 | No | Yes | 78% | 13.6 | 24% | NR | Some concerns |
|  |  |  |  |  |  |  |  |  |  |  |  |  |  |
| Derosier 2012 [4] | double‐blind, randomized, placebo‐controlled, parallel group trial | ICHD‐2 | Sumatriptan / Naproxen Sodium | Po | Eligible participants were 12 to 17 years old. In the double‐blind phase, subjects treated 1 moderate to severe migraine with either matching placebo or sumatriptan + naproxen sodium 10 mg + 60 mg, 30 mg + 180 mg, or 85 mg + 500 mg. | 490 | No | Yes | 59% | 14.7 | NR | Allowed | Low |
| Evers 2006 | Double‐blind, placebo‐controlled, cross‐over trial | ICHD‐2 | Zolmitriptan | Po | Ibuprofen 200 mg PO (children under 12) or 400 mg PO (adolescents) Zolmitriptan 2.5 mg PO | 29 | Yes | Yes | 56% | 13.9 | NR | NR | Some concerns |
| Fujita 2014 [6] | Randomized, double‐blind, placebo‐controlled, parallel group trial in Japan | ICHD‐2 | Sumatriptan | Po | Eligible patients were males and females 10–17 years old. Oral sumatriptan 25 mg (1 tablet and 1 matching placebo), sumatriptan 50 mg (2 tablets), or placebo (2 tablets) | 144 | Yes | Yes | 58% | 14.1 | 26% | NOT Allowed | Some concerns |
| Hämäläinen 1997a [7] | Randomized, double‐blind, placebo‐controlled, 3‐way cross‐over trial in the Greater Helsinki Area of Finland | IHS | Paracetamol Ibuprofen | Po | Each participant treated 1 of 3 migraine attacks with either oral paracetamol (15 mg/kg), oral ibuprofen (10 mg/kg), or placebo | 80 | Yes | Yes | 50% | 10.7 | 30% | NR | Some concerns |
| Hämäläinen 1997b [8] | Randomized, double‐blind, placebo‐controlled, 2‐way cross‐over trial | IHS | Sumatriptan | Po | Each participant treated 1 migraine attack with oral sumatriptan (50 mg for a body surface area of 0.75‐1.5 m2 (~6‐12 years of age) and 100 mg for a body surface area greater than 1.5 m2 (~>12 years of age) and 1 migraine attack with placebo | 23 | Yes | Yes | 52% | 12.3 | 29% | NR | Some concerns |
| Hämäläinen 1997c [9] | Randomized, double‐blind, placebo‐controlled, 4‐way cross‐over trial | IHS | Dihydroergotamine | Po | Dihydroergotamine (DHE) mesylate oral solution 2 mg/ml and placebo | 13 | Yes | Yes | 38% | 10.3 | 31% | NOT Allowed | High |
| Hämäläinen 2002 [10] | Randomized, double‐blind, placebo‐controlled, single attack cross‐over trial in Germany | IHS | Sumatriptan | IN | Each participant treated 1 migraine attack with nasal sumatriptan (10 mg) or placebo nasal spray. The second migraine was treated with the other medication | 59 | Yes | No | 54% | 9.7 | 24% | NOT Allowed | Some concerns |
| Ho 2012 [11] | Randomized, double‐blind, placebo‐controlled, parallel group trial at 191 sites (134 sites in the United States and 57 sites in Europe, India, and Canada) from December 2009 to April 2011 | ICHD‐2 | Rizatriptan | Po | Oral‐disintegrating tablet of rizatriptan 5 mg (< 40 kg) or 10 mg (≥ 40 kg) or placebo | 570 | Yes | Yes | 61% | NR | NR | NR | Some concerns |
| Lewis 2002 [12] | Randomized, double‐blind, placebo‐controlled, parallel‐group trial | IHS | Ibuprofen | Po | Each participant treated 1 migraine with liquid ibuprofen suspension (7.5 mg/kg) or placebo | 84 | Yes | No | 57% | 9.1 | 24% | Allowed (No change in prophylactic drug dose within 30 days) | Some concerns |
| Lewis 2007 [13] | Multicenter, randomized, double‐blind, placebo‐controlled, 2‐way, 2‐attack, cross‐over study | IHS | Zolmitriptan | IN | Adolescents of age 12‐17 years with a diagnosis of migraine with or without aura. Each participant treated 1 migraine attack with zolmitriptan 5 mg nasal spray and another with matching placebo within a 12‐week period | 171 | No | Yes | 57% | 14.2 | 16% | Allowed (No change in prophylactic drug dose within 30 days) | Some concerns |
| Linder 2008 [14] | Randomized, double‐blind, placebo‐controlled, parallel‐group multicenter trial from the United States (81 sites), Argentina (6 sites), Colombia (3 sites), and Mexico (3 sites) | IHS | Almotriptan | Po | Each participant treated 1 attack with either oral almotriptan (6.25 mg, 12.5 mg, or 25 mg) or placebo as soon as possible within 4 h after the onset of moderate to severe pain | 714 | No | Yes | 60% | 14.4 | NR | NR | Some concerns |
| Rothner 1997 [15] | Randomized, double‐blind, placebo‐controlled, parallel trial in the United States (44 sites) | IHS | Naratriptan | Po | Each participant treated 1 migraine with oral naratriptan (0.25 mg, 1.0 mg, or 2.5 mg) or placebo | 300 | No | Yes | 54% | 14.3 | NR | NR | High |
| Rothner 1999a [16] | Randomized, double‐blind, placebo‐controlled, parallel‐group 3‐attack trial from 62 centers in 7 countries | IHS | Sumatriptan | Po | Each participant treated 1 migraine with oral sumatriptan 25 mg, 50 mg, 100 mg, or placebo | 273 | No | Yes | 57% | 14.1 | NR | NR | High |
| Rothner 1999b [17] | Randomized, double‐blind, placebo‐controlled, parallel‐group trial in Canada (14 sites) | IHS | Sumatriptan | Po | Each participant treated 1 migraine with oral sumatriptan (50 mg for body weight 30‐50kg; 100 mg for body weight > 50kg) or placebo | 92 | No | Yes | 52% | 13.6 | NR | NR | High |
| Rothner 1999c [18] | Randomized, double‐blind, placebo‐controlled, parallel‐group trial from 18 centers in 8 countries | IHS | Sumatriptan | Po | Oral sumatriptan 50 mg (30 to 50 kg) or sumatriptan 100 mg (> 50 kg) versus placebo | 102 | No | Yes | 42% | 13.5 | NR | NR | Some concerns |
| Rothner 2006 [19] | Randomized, double‐blind, placebo‐controlled, parallel‐group trial from the United States (40 sites), Canada (10 sites), India (23 sites), Finland, Germany, and the United Kingdom | IHS | Zolmitriptan | Po | Each participant treated 1 migraine attack with oral zolmitriptan (2.5 mg, 5 mg, or 10 mg) or placebo no later than 1 h after the onset of moderate or severe headache | 696 | No | Yes | 59% | 14.2 | 13% | NR | Low |
| Ueberall 1999 [20] | Randomized, double‐blind, placebo‐controlled, 2‐way cross‐over trial | IHS | Sumatriptan | IN | Each participant treated 1 migraine with intranasal sumatriptan (20 mg) or placebo. The second migraine was treated with the other medication | 14 | Yes | No | 50% | 8.2 | 25% | NR | High |
| Visser 2004 [21] | Randomized, double‐blind, placebo‐controlled, parallel‐group single‐attack trial from 44 centers in the United States | IHS | Rizatriptan | Po | Each participant treated 1 migraine with oral rizatriptan (5 mg) or placebo within 30 minutes of onset | 476 | No | Yes | 55% | 14.2 | NR | NR | Some concerns |
| Winner 1997 [22] | Randomized, double‐blind, placebo‐controlled, 4‐period, outpatient, cross‐over, 4‐attack study | IHS | Sumatriptan | Po | Each participant treated up to 4 migraine attacks of moderate or severe intensity in a cross‐over fashion; 1 attack with placebo and 3 with sumatriptan 25 mg, 50 mg, or 100 mg (same dose for all 3 attacks) | 298 | No | Yes | 58% | 13.9 | 25% | NR | High |
| Winner 2000 [23] | Randomized, double‐blind, placebo‐controlled, single‐attack, parallel‐group study | IHS | Sumatriptan | IN | Each participant treated 1 migraine with nasal sumatriptan (5, 10, or 20 mg) or placebo. A second dose of the same nasal spray could be used 2‐24 h after the initial dose | 507 | No | Yes | 52% | 14.1 | 18% | NR | Low |
| Winner 2002 [24] | Randomized, double‐blind, placebo‐controlled, parallel‐group trial of oral rizatriptan | IHS | Rizatriptan | Po | Each participant was instructed to take the study medication (rizatriptan 5 mg or placebo) within 30 min of onset of a moderate or severe migraine | 296 | No | Yes | 54% | 14.0 | NR | NR | High |
| Winner 2006 [25] | Randomized, double‐blind, placebo‐controlled, parallel‐group, multicenter, single‐attack, outpatient study | IHS | Sumatriptan | IN | Sumatriptan 5 mg nasal spray; sumatriptan 20 mg nasal spray; or placebo | 731 | No | Yes | 55% | 14.3 | NR | NR | Low |
| Winner 2007 [26] | Randomized, double‐blind, parallel‐group, placebo‐controlled trial | ICHD‐2 | Eletriptan | Po | Eletriptan 40 mg PO, placebo taken within 4 h of headache onset | 274 | No | Yes | 57% | 14.0 | 30% | NR | Some concerns |
| Winner 2015 [27] | multi-centered, randomized, placebo-controlled, early intervention, cross-over study in the United States | ICHD‐2 | Sumatriptan/naproxen sodium | Po | Study medication was a fixed dose, single-tablet formulation containing 85 mg sumatriptan succinate, and 500 mg naproxen sodium. Subjects were trained to treat the headache in the mild phase within 1 hour of pain onset | 94 | No | Yes | 61% | 14.7 | NR | Allowed (No change in prophylactic drug dose within 60 days) | Low |
| Winner 2016 [28] | randomized, double-blind, placebo-controlled, four-arm parallel group study at 153 study centers in the US Latin America, and Europe between October 2010 and October 2013 | ICHD‐2 | Zolmitriptan | IN | Patients completed a 30-day run-in period to treat a single migraine attack with single-blind placebo nasal spray. Eligible patients, who had not responded to placebo, were randomized to one of three zolmitriptan nasal spray doses (5, 2.5, or 0.5 mg) or placebo | 721 | No | Yes | 64% | 14.3 | 22% | NR | Some concerns |
| Pavithra 2020 [29] | randomized, double-blind study in India between 20 May, 2017 and 22 March, 2018 | ICHD‐3 | Paracetamol, ibuprofen | Po | one group (n = 25) receiving oral paracetamol (15 mg/kg/dose) and the other group (n = 25) oral ibuprofen (10 mg/kg/dose), at home, during a single episode of acute migraine headache. | 43 | Yes | No | 48% | 9.9 | NR | NR | Some concerns |
| Yonker 2022 [30] | Phase 3, randomized, double-blind, placebo-controlled, multicenter crossover trial | ICHD‐3 | Zolmitriptan | IN | Patients <50 kg who were randomly allocated to ZNS were randomized to 5:1 to ZNS 2.5 or 1.0 mg; those ≥50 kg were randomized 5:1 to ZNS 5.0 or 2.5 mg. | 186 | Yes | No | 62% | 9.4 | 23% | Allowed (No change in prophylactic drug dose within 60 days) | High |
| IHS, international headache society; ICHD, International Classification of Headache Disorders; NR, not reported; Po, peros; IN, intranasal. | | | | | | | | | | | | |  |

**eTable 2:** SUCRA of pain freedom at 2 hours

| **Treatment** | **SUCRA, n%** |
| --- | --- |
| dihydroergotamine | 88.9 |
| sumatriptan/naproxen sodium | 84.5 |
| ibuprofen | 83.2 |
| zolmitriptan NS | 70.4 |
| ibuprofen SUS | 67.7 |
| acetaminophen | 54.4 |
| sumatriptan NS | 53.2 |
| eletriptan | 47.3 |
| rizatriptan | 43.3 |
| zolmitriptan | 37.3 |
| almotriptan | 23.0 |
| naratriptan | 20.3 |
| sumatriptan | 16.4 |
| placebo | 9.5 |

SUCRA values vary from 0 to 1, whereas 1 means the treatment might be the best and 0 the worst.

**eTable 3:** SUCRA of adverse events

| **Treatment** | **SUCRA, n%** |
| --- | --- |
| placebo | 87.3 |
| acetaminophen | 74.6 |
| rizatriptan | 68.8 |
| ibuprofen | 53.6 |
| zolmitriptan NS | 50.8 |
| almotriptan | 50.5 |
| eletriptan | 49.4 |
| sumatriptan/naproxen sodium | 45.4 |
| naratriptan | 38.8 |
| sumatriptan | 34.9 |
| almotriptan | 23.5 |
| sumatriptan NS | 22.4 |

SUCRA values vary from 0 to 1, whereas 1 means the treatment might be the best and 0 the worst.

**eTable 4:** SUCRA of pain relief at 2 hours

| **Treatment** | **SUCRA, n%** |
| --- | --- |
| dihydroergotamine | 86.6 |
| ibuprofen | 77.5 |
| sumatriptan NS | 73.9 |
| ibuprofen SUS | 66.1 |
| almotriptan | 55.4 |
| acetaminophen | 54.5 |
| zolmitriptan NS | 53.5 |
| rizatriptan | 50.1 |
| zolmitriptan | 47.7 |
| eletriptan | 25.0 |
| sumatriptan | 24.6 |
| placebo | 22.2 |
| naratriptan | 13.1 |

SUCRA values vary from 0 to 1, whereas 1 means the treatment might be the best and 0 the worst.

**eTable 5:** SUCRA of use of rescue drugs from 2 to 24 hours

| **Treatment** | **SUCRA, n%** |
| --- | --- |
| zolmitriptan | 88.2 |
| sumatriptan/naproxen sodium | 81.8 |
| ibuprofen | 77.6 |
| almotriptan | 57.0 |
| sumatriptan NS | 51.2 |
| rizatriptan | 51.0 |
| acetaminophen | 49.3 |
| sumatriptan | 39.5 |
| eletriptan | 38.6 |
| zolmitriptan NS | 28.5 |
| naratriptan | 25.9 |
| placebo | 11.4 |

SUCRA values vary from 0 to 1, whereas 1 means the treatment might be the best and 0 the worst.

**eTable 6:** SUCRA of pain freedom from 2 to 24 hours

| **Treatment** | **SUCRA, n%** |
| --- | --- |
| sumatriptan/naproxen sodium | 75.5 |
| ibuprofen | 70.9 |
| acetaminophen | 49.5 |
| rizatriptan | 49.0 |
| placebo | 5.1 |

SUCRA values vary from 0 to 1, whereas 1 means the treatment might be the best and 0 the worst.

**eTable 7:** League table of pain relief at 2 hours

| **acetaminophen** |  |  |  | 0.57(0.18,1.80) |  |  |  |  |  |  |  | 1.95(0.52,7.34) |
| --- | --- | --- | --- | --- | --- | --- | --- | --- | --- | --- | --- | --- |
| 0.99 (0.15,6.39) | **almotriptan** |  |  |  |  |  |  |  |  |  |  | 1.92(0.66,5.54) |
| 0.27 (0.02,3.82) | 0.27 (0.02,3.81) | **dihydroergotamine** |  |  |  |  |  |  |  |  |  | 7.00(0.81,60.02) |
| 2.11 (0.32,13.98) | 2.13 (0.33,13.91) | 7.74 (0.55,109.54) | **eletriptan** |  |  |  |  |  |  |  |  | 0.90(0.29,2.73) |
| 0.60 (0.18,2.01) | 0.60 (0.11,3.25) | 2.18 (0.18,27.13) | 0.28 (0.05,1.57) | **ibuprofen** |  |  |  |  |  | 1.16(0.26,5.02) |  | **4.10(1.50,11.17)** |
| 0.72 (0.09,5.60) | 0.73 (0.09,5.57) | 2.64 (0.17,41.99) | 0.34 (0.04,2.69) | 1.21 (0.18,8.04) | **ibuprofen SUS** |  |  |  |  |  |  | 2.64(0.67,10.33) |
| 3.14 (0.47,21.09) | 3.16 (0.48,20.98) | 11.50 (0.80,164.54) | 1.49 (0.22,10.13) | 5.27 (0.93,29.92) | 4.35 (0.54,34.77) | **naratriptan** |  |  |  |  |  | 0.60(0.19,1.89) |
| 1.15 (0.26,5.06) | 1.15 (0.27,5.02) | 4.20 (0.39,45.31) | 0.54 (0.12,2.44) | 1.92 (0.55,6.79) | 1.59 (0.29,8.71) | 0.37 (0.08,1.68) | **rizatriptan** |  |  |  |  | 1.64(0.95,2.83) |
| 1.90 (0.44,8.19) | 1.91 (0.45,8.13) | 6.96 (0.65,74.00) | 0.90 (0.20,3.96) | 3.19 (0.93,10.96) | 2.63 (0.49,14.15) | 0.61 (0.13,2.72) | 1.66 (0.67,4.12) | **sumatriptan** |  |  |  | 1.00(0.58,1.70) |
| 0.68 (0.16,2.99) | 0.69 (0.16,2.98) | 2.50 (0.23,26.91) | 0.32 (0.07,1.45) | 1.14 (0.33,4.00) | 0.95 (0.17,5.17) | 0.22 (0.05,1.00) | 0.59 (0.23,1.52) | 0.36 (0.15,0.89) | **sumatriptan NS** |  |  | **2.56(1.50,4.35)** |
| 1.18 (0.25,5.63) | 1.19 (0.23,6.21) | 4.33 (0.36,52.51) | 0.56 (0.10,3.01) | 1.98 (0.56,7.08) | 1.64 (0.25,10.54) | 0.38 (0.07,2.06) | 1.03 (0.31,3.45) | 0.62 (0.19,2.03) | 1.73 (0.53,5.66) | **zolmitriptan** |  | 1.48(0.62,3.52) |
| 1.05 (0.20,5.35) | 1.05 (0.21,5.32) | 3.83 (0.32,45.45) | 0.49 (0.09,2.58) | 1.75 (0.42,7.36) | 1.45 (0.23,9.06) | 0.33 (0.06,1.77) | 0.91 (0.28,2.93) | 0.55 (0.18,1.72) | 1.53 (0.48,4.91) | 0.88 (0.22,3.55) | **zolmitriptan NS** | 1.82(0.83,4.01) |
| 1.91 (0.51,7.19) | 1.92 (0.52,7.11) | 7.00 (0.71,68.55) | 0.90 (0.24,3.48) | **3.21 (1.10,9.34)** | 2.65 (0.55,12.65) | 0.61 (0.15,2.39) | 1.67 (0.85,3.25) | 1.01 (0.54,1.87) | **2.80 (1.44,5.45)** | 1.62 (0.59,4.44) | 1.83 (0.70,4.75) | **placebo** |

Pairwise (upper-right portion) and network (lower-left portion) meta-analysis results are presented as estimated effect sizes for pain freedom after 1 hour. For the result, outcomes are expressed as odds ratios (OR) with 95% confidence interval (CI) (OR of > 1 indicated that the treatment specified in the row got more improvement than that specified in the column), 0 < OR < 1, the opposite. For the network meta-analysis, OR of > 1 indicates that the treatment specified in the column got better improvement than that specified in the row, 0 < OR < 1, the opposite. 95% CI that did not contain one was considered to have a statistical difference. Bold results indicate statistical significance. Marked with * indicated a significant difference between direct and mixed comparisons. NS, nose spray; SUS, suspension.

**eTable 8:** League table of use of rescue drugs from 2 to 24 hours

| **acetaminophen** | 1.10 (0.21,5.52) |  | 1.54 (0.38,6.33) |  |  |  |  |  |  |  | 0.64(0.19,2.17) |
| --- | --- | --- | --- | --- | --- | --- | --- | --- | --- | --- | --- |
| 1.12 (0.26,4.88) | **almotriptan** |  |  |  |  |  |  |  |  |  | 0.55(0.23,1.31) |
| 0.84 (0.22,3.28) | 0.75 (0.25,2.29) | **eletriptan** |  |  |  |  |  |  |  |  | 0.73(0.38,1.40) |
| 1.69 (0.46,6.22) | 1.52 (0.40,5.80) | 2.01 (0.60,6.75) | **ibuprofen** |  |  |  |  |  | 1.65 (0.34,9.81) |  | 0.36(0.13,0.98) |
| 0.68 (0.17,2.83) | 0.61 (0.19,2.00) | 0.81 (0.29,2.29) | 0.40 (0.11,1.45) | **naratriptan** |  |  |  |  |  |  | 0.90(0.41,1.94) |
| 0.97 (0.28,3.38) | 0.87 (0.33,2.29) | 1.16 (0.53,2.52) | 0.57 (0.20,1.69) | 1.43 (0.59,3.44) | **rizatriptan** |  |  |  |  |  | 0.63(0.43,0.92) |
| 0.85 (0.24,3.01) | 0.76 (0.28,2.05) | 1.01 (0.45,2.27) | 0.50 (0.17,1.51) | 1.24 (0.50,3.08) | 0.87 (0.48,1.58) | **sumatriptan** |  |  |  |  | 0.72(0.46,1.12) |
| 0.97 (0.28,3.33) | 0.87 (0.34,2.25) | 1.16 (0.54,2.46) | 0.57 (0.20,1.66) | 1.42 (0.60,3.36) | 1.00 (0.60,1.65) | 1.15 (0.65,2.01) | **sumatriptan NS** |  |  |  | 0.63(0.46,0.87) |
| 1.70 (0.44,6.54) | 1.53 (0.51,4.56) | 2.02 (0.80,5.14) | 1.00 (0.30,3.33) | 2.49 (0.90,6.91) | 1.75 (0.82,3.72) | 2.00 (0.91,4.40) | 1.75 (0.84,3.63) | **sumatriptan/ naproxen sodium** |  |  | 0.36(0.19,0.67) |
| 2.98 (0.41,21.72) | 2.67 (0.40,17.68) | 3.54 (0.58,21.43) | 1.76 (0.30,10.32) | 4.36 (0.69,27.66) | 3.06 (0.55,17.03) | 3.51 (0.62,19.79) | 3.06 (0.56,16.85) | 1.75 (0.29,10.50) | **zolmitriptan** |  | 0.19(0.03,1.06) |
| 0.75 (0.21,2.67) | 0.67 (0.24,1.83) | 0.89 (0.39,2.02) | 0.44 (0.14,1.34) | 1.09 (0.43,2.75) | 0.77 (0.41,1.43) | 0.88 (0.46,1.69) | 0.77 (0.42,1.39) | 0.44 (0.20,0.98) | 0.25 (0.04,1.42) | **zolmitriptan NS** | 0.82(0.51,1.30) |
| 0.62 (0.19,2.01) | 0.55 (0.23,1.34) | 0.73 (0.37,1.43) | 0.36 (0.13,1.00) | 0.90 (0.41,1.98) | 0.63 (0.43,0.94) | 0.72 (0.46,1.14) | 0.63 (0.45,0.89) | 0.36 (0.19,0.69) | 0.21 (0.04,1.10) | 0.83 (0.51,1.33) | **placebo** |

odds ratios (OR) with 95% confidence interval (CI) (OR of > 1 indicated that the treatment specified in the row got more improvement than that specified in the column), 0 < OR < 1, the opposite. For the network meta-analysis, OR of > 1 indicated that the treatment specified in the column got better improvement than that specified in the row, 0 < OR < 1, the opposite. 95% CI that did not contain one was considered to have a statistical difference. Bold results indicated statistical significance. Marked with * indicated a significant difference between direct and mixed comparisons. ^ indicated a significant difference between the basic model and sensitivity analysis. NS, nose spray; SUS, suspension.

**eTable 9:** League table of pain freedom from 2 to 24 hours

| **acetaminophen** | 0.71(0.31,1.64) |  |  | 1.71(0.63,4.58) |
| --- | --- | --- | --- | --- |
| 0.72 (0.29,1.80) | **ibuprofen** |  |  | ***2.70(1.02,7.13)** |
| 1.05 (0.32,3.49) | 1.46 (0.44,4.81) | **rizatriptan** |  | ***1.73(1.15,2.61)** |
| 0.79 (0.24,2.61) | 1.10 (0.34,3.58) | 0.75 (0.33,1.70) | **sumatriptan/ naproxen sodium** | **2.31(1.43,3.76)** |
| 1.83 (0.64,5.21) | 2.54 (0.90,7.16) | 1.74 (0.97,3.12) | **2.31 (1.31,4.07)** | **placebo** |

odds ratios (OR) with 95% confidence interval (CI) (OR of > 1 indicated that the treatment specified in the row got more improvement than that specified in the column), 0 < OR < 1, the opposite. For the network meta-analysis, OR of > 1 indicated that the treatment specified in the column got better improvement than that specified in the row, 0 < OR < 1, the opposite. 95% CI that did not contain one was considered to have a statistical difference. Bold results indicated statistical significance. Marked with * indicated a significant difference between direct and mixed comparisons. ^ indicated a significant difference between the basic model and sensitivity analysis.

**eTable 10:** Sensitivity analysis of pain freedom at 2 hours

| **acetaminophen** |  |  |  |  |  |  |  |  |  |  |  |  |
| --- | --- | --- | --- | --- | --- | --- | --- | --- | --- | --- | --- | --- |
| 1.53 (0.64,3.65) | **almotriptan** |  |  |  |  |  |  |  |  |  |  |  |
| 1.12 (0.41,3.08) | 0.74 (0.36,1.51) | **eletriptan** |  |  |  |  |  |  |  |  |  |  |
| 0.62 (0.31,1.25) | 0.41 (0.19,0.87) | 0.55 (0.22,1.38) | **ibuprofen** |  |  |  |  |  |  |  |  |  |
| 0.77 (0.23,2.62) | 0.50 (0.19,1.37) | 0.69 (0.22,2.10) | 1.24 (0.40,3.91) | **ibuprofen SUS** |  |  |  |  |  |  |  |  |
| 1.65 (0.60,4.56) | 1.08 (0.52,2.24) | 1.47 (0.61,3.57) | **2.66 (1.06,6.70)** | 2.14 (0.70,6.59) | **naratriptan** |  |  |  |  |  |  |  |
| 1.13 (0.50,2.58) | 0.74 (0.48,1.13) | 1.01 (0.52,1.95) | 1.82 (0.90,3.70) | 1.47 (0.56,3.82) | 0.69 (0.35,1.34) | **rizatriptan** |  |  |  |  |  |  |
| 1.70 (0.70,4.13) | 1.11 (0.65,1.90) | 1.52 (0.73,3.16) | **2.75 (1.26,5.98)** | 2.21 (0.81,6.06) | 1.03 (0.49,2.18) | 1.51 (0.96,2.37) | **sumatriptan** |  |  |  |  |  |
| 1.06 (0.46,2.43) | 0.70 (0.45,1.07) | 0.95 (0.49,1.83) | 1.71 (0.84,3.48) | 1.38 (0.53,3.59) | 0.64 (0.33,1.26) | 0.94 (0.68,1.30) | 0.62 (0.40,0.98) | **sumatriptan NS** |  |  |  |  |
| 0.61 (0.25,1.52) | 0.40 (0.23,0.71) | 0.55 (0.26,1.17) | 0.99 (0.44,2.21) | 0.80 (0.29,2.23) | 0.37 (0.17,0.81) | 0.54 (0.33,0.89) | 0.36 (0.20,0.65) | *0.58 (0.35,0.95) | **sumatriptan/ naproxen sodium** |  |  |  |
| 1.28 (0.55,2.98) | 0.84 (0.48,1.44) | 1.14 (0.54,2.39) | **2.06 (1.02,4.17)** | 1.66 (0.60,4.57) | 0.77 (0.36,1.65) | 1.13 (0.70,1.81) | 0.75 (0.42,1.33) | 1.20 (0.75,1.93) | **2.08 (1.13,3.80)** | **zolmitriptan** |  |  |
| 0.84 (0.36,1.95) | 0.55 (0.35,0.87) | 0.75 (0.38,1.48) | 1.35 (0.65,2.81) | 1.09 (0.41,2.88) | 0.51 (0.25,1.02) | 0.74 (0.51,1.07) | 0.49 (0.30,0.80) | 0.79 (0.55,1.14) | 1.36 (0.80,2.31) | 0.66 (0.40,1.09) | **zolmitriptan NS** |  |
| 1.79 (0.81,3.96) | 1.17 (0.82,1.68) | 1.59 (0.86,2.96) | **2.88 (1.47,5.64)** | 2.32 (0.92,5.87) | 1.08 (0.57,2.04) | **1.58 (1.26,1.99)** | 1.05 (0.71,1.56) | **1.68 (1.34,2.12)** | **2.91 (1.87,4.53)** | 1.40 (0.93,2.12) | **2.13 (1.60,2.85)** | **placebo** |

For the result, outcomes are expressed as odds ratios (OR) with 95% confidence interval (CI) (OR of > 1 indicated that the treatment specified in the row got more improvement than that specified in the column), 0 < OR < 1, the opposite. For the network meta-analysis, OR of > 1 indicated that the treatment specified in the column got better improvement than that specified in the row, 0 < OR < 1, the opposite. 95% CI that did not contain one was considered to have a statistical difference. Bold results indicated statistical significance. Marked with * indicated a significant difference between the basic model and sensitivity analysis.

**eTable 11:** Sensitivity analysis of adverse events

| **acetaminophen** |  |  |  |  |  |  |  |  |  |  |  |
| --- | --- | --- | --- | --- | --- | --- | --- | --- | --- | --- | --- |
| 0.52 (0.06,4.89) | **almotriptan** |  |  |  |  |  |  |  |  |  |  |
| 0.52 (0.06,4.68) | 1.00 (0.16,6.30) | **eletriptan** |  |  |  |  |  |  |  |  |  |
| 0.56 (0.09,3.40) | 1.08 (0.18,6.46) | 1.08 (0.19,6.12) | **ibuprofen** |  |  |  |  |  |  |  |  |
| 0.41 (0.04,3.83) | 0.79 (0.12,5.18) | 0.79 (0.13,4.92) | 0.73 (0.12,4.33) | **naratriptan** |  |  |  |  |  |  |  |
| 0.60 (0.08,4.30) | 1.16 (0.24,5.49) | 1.16 (0.26,5.15) | 1.07 (0.26,4.50) | 1.46 (0.31,6.83) | **rizatriptan** |  |  |  |  |  |  |
| 0.36 (0.05,2.45) | 0.70 (0.16,3.08) | 0.70 (0.17,2.88) | 0.65 (0.17,2.50) | 0.88 (0.20,3.83) | 0.60 (0.21,1.69) | **sumatriptan** |  |  |  |  |  |
| 0.32 (0.05,2.13) | 0.62 (0.14,2.67) | 0.62 (0.15,2.50) | 0.57 (0.15,2.16) | 0.78 (0.18,3.32) | 0.53 (0.20,1.43) | 0.89 (0.37,2.13) | **sumatriptan NS** |  |  |  |  |
| 0.47 (0.06,3.85) | 0.91 (0.16,5.08) | 0.91 (0.17,4.80) | 0.84 (0.17,4.20) | 1.15 (0.21,6.33) | 0.78 (0.21,2.97) | 1.30 (0.37,4.60) | 1.47 (0.43,5.02) | **sumatriptan NS** |  |  |  |
| 0.31 (0.04,2.22) | 0.60 (0.11,3.15) | 0.60 (0.12,2.97) | 0.56 (0.15,2.02) | 0.76 (0.15,3.93) | 0.52 (0.15,1.84) | 0.86 (0.27,2.78) | 0.97 (0.31,3.04) | 0.66 (0.15,2.85) | **zolmitriptan** |  |  |
| 0.52 (0.07,3.67) | 1.01 (0.22,4.67) | 1.01 (0.23,4.37) | 0.93 (0.23,3.80) | 1.27 (0.28,5.80) | 0.87 (0.29,2.60) | 1.45 (0.54,3.87) | 1.63 (0.63,4.21) | 1.11 (0.30,4.14) | 1.68 (0.49,5.74) | **zolmitriptan NS** |  |
| 0.98 (0.16,5.91) | 1.88 (0.49,7.17) | 1.88 (0.53,6.66) | 1.74 (0.53,5.72) | 2.38 (0.63,8.90) | 1.62 (0.73,3.60) | **2.70 (1.42,5.14)** | **3.04 (1.69,5.46)** | 2.07 (0.70,6.12) | **3.13 (1.18,8.33)** | 1.87 (0.88,3.94) | **placebo** |

interval (CI) (OR of > 1 indicated that the treatment specified in the row got more improvement than that specified in the column), 0 < OR < 1, the opposite. For the network meta-analysis, OR of > 1 indicated that the treatment specified in the column got better improvement than that specified in the row, 0 < OR < 1, the opposite. 95% CI that did not contain one was considered to have a statistical difference. Bold results indicated statistical significance. Marked with ^ indicated a significant difference between the basic model and sensitivity analysis. NS, nose spray.

**eTable 12:** Subgroup analysis of pain freedom at 2 hours in Children

| **acetaminophen** |  |  |  |  |  |  |
| --- | --- | --- | --- | --- | --- | --- |
| 0.68 (0.21,2.23) | **ibuprofen** |  |  |  |  |  |
| 0.85 (0.10,7.35) | 1.25 (0.14,10.77) | **ibuprofen SUS** |  |  |  |  |
| 1.31 (0.17,9.95) | 1.91 (0.25,14.58) | 1.53 (0.23,10.35) | **rizatriptan** |  |  |  |
| 0.54 (0.07,4.19) | 0.79 (0.10,6.15) | 0.63 (0.09,4.37) | 0.41 (0.07,2.48) | **sumatriptan NS** |  |  |
| 1.19 (0.16,8.91) | 1.73 (0.23,13.07) | 1.39 (0.21,9.27) | 0.91 (0.16,5.24) | 2.21 (0.37,13.19) | **zolmitriptan NS** |  |
| 1.98 (0.40,9.78) | 2.90 (0.58,14.35) | 2.32 (0.55,9.83) | 1.51 (0.43,5.29) | **3.69 (1.01,13.47)** | 1.67 (0.49,5.73) | **placebo** |

interval (CI) (OR of > 1 indicated that the treatment specified in the row got more improvement than that specified in the column), 0 < OR < 1, the opposite. For the network meta-analysis, OR of > 1 indicated that the treatment specified in the column got better improvement than that specified in the row, 0 < OR < 1, the opposite. 95% CI that did not contain one was considered to have a statistical difference. Bold results indicated statistical significance. NS, nose spray; SUS, suspension.

**eTable 13:** Subgroup analysis of pain freedom at 2 hours in Adolescents

| **almotriptan** |  |  |  |  |  |  |  |  |  |
| --- | --- | --- | --- | --- | --- | --- | --- | --- | --- |
| 0.74 (0.36,1.51) | **eletriptan** |  |  |  |  |  |  |  |  |
| 1.08 (0.52,2.24) | 1.47 (0.61,3.57) | **naratriptan** |  |  |  |  |  |  |  |
| 0.82 (0.53,1.26) | 1.11 (0.57,2.16) | 0.76 (0.38,1.49) | **rizatriptan** |  |  |  |  |  |  |
| 1.01 (0.57,1.78) | 1.37 (0.64,2.93) | 0.93 (0.43,2.02) | 1.23 (0.75,2.03) | **sumatriptan** |  |  |  |  |  |
| 0.73 (0.47,1.13) | 0.99 (0.51,1.94) | 0.67 (0.34,1.34) | 0.89 (0.63,1.26) | 0.72 (0.43,1.20) | **sumatriptan NS** |  |  |  |  |
| 0.40 (0.23,0.71) | 0.55 (0.26,1.17) | 0.37 (0.17,0.81) | 0.49 (0.30,0.81) | 0.40 (0.21,0.75) | 0.55 (0.33,0.92) | **sumatriptan/ naproxen sodium** |  |  |  |
| 1.00 (0.57,1.77) | 1.36 (0.64,2.92) | 0.93 (0.43,2.01) | 1.22 (0.74,2.02) | 0.99 (0.53,1.85) | 1.37 (0.82,2.29) | 2.49 (1.33,4.65) | **zolmitriptan** |  |  |
| 0.50 (0.30,0.82) | 0.68 (0.33,1.38) | 0.46 (0.22,0.95) | 0.61 (0.40,0.92) | 0.50 (0.28,0.86) | 0.68 (0.45,1.05) | 1.24 (0.71,2.17) | 0.50 (0.28,0.87) | **zolmitriptan NS** |  |
| 1.17 (0.82,1.68) | 1.59 (0.86,2.96) | 1.08 (0.57,2.04) | **1.43 (1.13,1.81)** | 1.16 (0.75,1.81) | **1.61 (1.24,2.08)** | **2.91 (1.87,4.53)** | 1.17 (0.75,1.82) | **2.35 (1.67,3.31)** | **placebo** |

interval (CI) (OR of > 1 indicated that the treatment specified in the row got more improvement than that specified in the column), 0 < OR < 1, the opposite. For the network meta-analysis, OR of > 1 indicated that the treatment specified in the column got better improvement than that specified in the row, 0 < OR < 1, the opposite. 95% CI that did not contain one was considered to have a statistical difference. Bold results indicated statistical significance. NS, nose spray; SUS, suspension.

**eTable 14:** Subgroup analysis of oral pharmacological agents

| **acetaminophen** |  |  |  |  |  |  |  |  |  |  |
| --- | --- | --- | --- | --- | --- | --- | --- | --- | --- | --- |
| 1.53 (0.64,3.65) | **almotriptan** |  |  |  |  |  |  |  |  |  |
| 0.23 (0.02,2.71) | 0.15 (0.01,1.60) | **dihydroergotamine** |  |  |  |  |  |  |  |  |
| 1.12 (0.41,3.08) | 0.74 (0.36,1.51) | 4.93 (0.44,55.89) | **eletriptan** |  |  |  |  |  |  |  |
| 0.62 (0.31,1.25) | 0.41 (0.19,0.87) | 2.72 (0.24,31.28) | 0.55 (0.22,1.38) | **ibuprofen** |  |  |  |  |  |  |
| 1.65 (0.60,4.56) | 1.08 (0.52,2.24) | 7.25 (0.64,82.44) | 1.47 (0.61,3.57) | **2.66 (1.06,6.70)** | **naratriptan** |  |  |  |  |  |
| 1.18 (0.52,2.69) | 0.78 (0.51,1.17) | 5.20 (0.49,54.85) | 1.05 (0.55,2.03) | 1.91 (0.95,3.85) | 0.72 (0.37,1.40) | **rizatriptan** |  |  |  |  |
| 1.67 (0.70,4.02) | 1.10 (0.65,1.84) | 7.35 (0.68,79.11) | 1.49 (0.72,3.07) | **2.70 (1.25,5.81)** | 1.01 (0.49,2.11) | 1.41 (0.92,2.16) | **sumatriptan** |  |  |  |
| 0.61 (0.25,1.53) | 0.40 (0.23,0.71) | 2.70 (0.25,29.40) | 0.55 (0.26,1.17) | 0.99 (0.44,2.21) | 0.37 (0.17,0.81) | 0.52 (0.32,0.85) | 0.37 (0.21,0.65) | **sumatriptan/ naproxen sodium** |  |  |
| 1.28 (0.55,2.98) | 0.84 (0.48,1.44) | 5.61 (0.52,60.73) | 1.14 (0.54,2.39) | **2.06 (1.02,4.17)** | 0.77 (0.36,1.65) | 1.08 (0.68,1.71) | 0.76 (0.44,1.33) | 2.08 (1.13,3.80) | **zolmitriptan** |  |
| 1.79 (0.81,3.96) | 1.17 (0.82,1.68) | 7.86 (0.75,82.10) | 1.59 (0.86,2.96) | **2.88 (1.47,5.64)** | 1.08 (0.57,2.04) | **1.51 (1.23,1.86)** | 1.07 (0.74,1.55) | **2.91 (1.87,4.53)** | 1.40 (0.93,2.12) | **placebo** |

interval (CI) (OR of > 1 indicated that the treatment specified in the row got more improvement than that specified in the column), 0 < OR < 1, the opposite. For the network meta-analysis, OR of > 1 indicated that the treatment specified in the column got better improvement than that specified in the row, 0 < OR < 1, the opposite. 95% CI that did not contain one was considered to have a statistical difference. Bold results indicated statistical significance. NS, nose spray; SUS, suspension.

**eTable15:** Design-by-treatment interaction model for inconsistency

| **design-by-treatment inconsistency** | **chi2** | **Prob>chi2** |
| --- | --- | --- |
| Pain freedom at 2 hours | 8.66 | 0.05 |
| Pain relief at 2 hours | 2.14 | 0.54 |
| Use of rescue drugs from 2 to 24 hours | 0.10 | 0.75 |
| Pain freedom from two to 24 hours | 0.29 | 0.59 |
| Any adverse events | 0.43 | 0.80 |

**eTable 16:** Significant loop-specific inconsistencies of network meta-analysis

| **loop inconsistency** | **Ratio of odds ratios** | **95% confidence interval** | **Tau^2^** | **P** |
| --- | --- | --- | --- | --- |
| **Pain freedom at 2 hours** |  |  |  |  |
| acetaminophen-ibuprofen-placebo | 1.275 | 0.00, 3.02 | 0.000 | 0.153 |
| ibuprofen-zolmitriptan-placebo | 0.902 | 0.00, 2.35 | 0.000 | 0.221 |
| **Pain relief at 2 hours** |  |  |  |  |
| ibuprofen-zolmitriptan-placebo | 1.09 | 0.00, 2.54 | 0.000 | 0.138 |
| acetaminophen-ibuprofen-placebo | 0.26 | 0.00, 2.47 | 0.000 | 0.238 |
| **Use of rescue drugs from 2 to 24 hours** |  |  |  |  |
| acetaminophen-ibuprofen-placebo | 1.163 | 0.00, 2.61 | 0.000 | 0.779 |
| ibuprofen-zolmitriptan-placebo | 1.427 | 0.00, 3.23 | 0.000 | 0.819 |
| **Pain freedom from two to 24 hours** |  |  |  |  |
| acetaminophen-ibuprofen-placebo | 0.638 | 0.00, 3.04 | 0.000 | 0.603 |
| **Adverse events** |  |  |  |  |
| ibuprofen-zolmitriptan-placebo | 0.832 | 0.00, 2.72 | 0.000 | 0.389 |
| acetaminophen-ibuprofen-placebo | 0.787 | 0.00, 3.37 | 0.000 | 0.598 |

**eTable 17:** Significant side-splitting inconsistencies of network meta-analysis

| **Side** | **Direct** | | **Indirect** | | **Difference** | | **P>z** | **Tau** | **Treatments used** |
| --- | --- | --- | --- | --- | --- | --- | --- | --- | --- |
|  | **Coefficient** | **SE** | **Coefficient** | **SE** | **Coefficient** | **SE** |  |  |  |
| **Pain freedom at 2 hours** | | | | | | | | | |
| A vs. E | 0.52 | 0.37 | -0.09 | 1.33 | 0.61 | 1.38 | 0.66 | 0.20 | A: acetaminophen |
| A vs. N | -0.49 | 0.47 | -0.87 | 0.82 | 0.38 | 0.95 | 0.69 | 0.21 | B: almotriptan |
| B vs. N | -0.16 | 0.18 | -1.08 | 130.14 | 0.92 | 130.14 | 0.99 | 0.21 | C: dihydroergotamine |
| C vs. N | -2.06 | 1.2 | -0.63 | 146.06 | -1.44 | 146.07 | 0.99 | 0.20 | D: eletriptan |
| D vs. N | -0.47 | 0.32 | -1.11 | 162.54 | 0.65 | 162.54 | 1 | 0.20 | E: ibuprofen |
| E vs. L | -0.14 | 0.53 | -1.23 | 0.49 | 1.09 | 0.72 | 0.13 | 0.21 | F: ibuprofen SUS |
| E vs. N | -1.62 | 0.4 | 1.12 | 0.9 | -2.74 | 1.04 | 0.01 | 0.21 | G: naratriptan |
| F vs. N | -0.84 | 0.47 | -1.05 | 131.99 | 0.21 | 131.99 | 1 | 0.20 | H: rizatriptan |
| G vs. N | -0.08 | 0.32 | -1.12 | 150.89 | 1.04 | 150.89 | 0.99 | 0.14 | I: sumatriptan |
| H vs. N | -0.41 | 0.11 | -1.15 | 69.13 | 0.74 | 69.13 | 0.99 | 0.14 | J: sumatriptan/naproxen sodium |
| I vs. N | -0.07 | 0.19 | -1.18 | 66.08 | 1.11 | 66.08 | 0.99 | 0.20 | K: sumatriptan NS |
| J vs. N | -1.07 | 0.23 | -0.51 | 101.43 | -0.56 | 101.43 | 1 | 0.20 | L: zolmitriptan |
| K vs. N | -0.52 | 0.12 | -1.12 | 55.98 | 0.59 | 55.98 | 0.99 | 0.20 | M: zolmitriptan NS |
| L vs. N | -0.31 | 0.22 | -0.93 | 1.35 | 0.61 | 1.39 | 0.66 | 0.20 | N: placebo |
| M vs. N | -0.76 | 0.15 | -1.09 | 82.24 | 0.33 | 82.24 | 1.00 | 0.20 |  |
| **Any adverse events** | | | | | | | | | |
| A vs. D | 0.34 | 0.98 | 1.93 | 2.25 | -1.59 | 2.42 | 0.51 | 0.57 | A: acetaminophen |
| A vs. L | 0.26 | 0.98 | -1.34 | 2.24 | 1.60 | 2.41 | 0.51 | 0.56 | B: almotriptan |
| B vs. L | -0.63 | 0.66 | 0.43 | 217.70 | -1.06 | 217.70 | 1.00 | 0.56 | C: eletriptan |
| C vs. L | -0.63 | 0.62 | 0.11 | 131.82 | -0.75 | 131.82 | 1.00 | 0.56 | D: ibuprofen |
| D vs. J | 0.33 | 0.81 | 1.06 | 1.10 | -0.74 | 1.36 | 0.59 | 0.57 | E: naratriptan |
| D vs. L | -0.49 | 0.65 | -1.03 | 1.90 | 0.53 | 2.04 | 0.80 | 0.58 | F: rizatriptan |
| E vs. L | -0.87 | 0.65 | 0.54 | 138.46 | -1.41 | 138.46 | 0.99 | 0.56 | G: sumatriptan |
| F vs. L | -0.31 | 0.33 | 0.04 | 80.41 | -0.36 | 80.41 | 1.00 | 0.56 | H: sumatriptan NS |
| G vs. L | -0.89 | 0.30 | 0.22 | 61.41 | -1.11 | 61.41 | 0.99 | 0.56 | I: sumatriptan/naproxen sodium |
| H vs. L | -1.11 | 0.29 | 0.32 | 59.48 | -1.43 | 59.48 | 0.98 | 0.56 | J: zolmitriptan |
| I vs. L | -0.72 | 0.54 | 0.48 | 149.68 | -1.20 | 149.69 | 0.99 | 0.56 | K: zolmitriptan NS |
| J vs. L | -1.22 | 0.50 | 0.39 | 2.36 | -1.61 | 2.43 | 0.51 | 0.57 | L: placebo |
| K vs. L | -0.63 | 0.37 | 0.13 | 101.16 | -0.75 | 101.16 | 0.99 | 0.56 |  |
| **Pain relief at 2 hours** | | | | | | | | | |
| A vs. E | 0.54 | 0.67 | 0.29 | 2.13 | 0.26 | 2.24 | 0.91 | 0.68 | A: acetaminophen |
| A vs. M | -0.67 | 0.80 | -0.59 | 1.37 | -0.08 | 1.59 | 0.96 | 0.67 | B: almotriptan |
| B vs. M | -0.65 | 0.67 | -0.97 | 134.33 | 0.32 | 134.33 | 1.00 | 0.64 | C: dihydroergotamine |
| C vs. M | -1.95 | 1.16 | -1.04 | 130.65 | -0.90 | 130.66 | 0.99 | 0.64 | D: eletriptan |
| D vs. M | 0.10 | 0.69 | -1.30 | 128.21 | 1.40 | 128.21 | 0.99 | 0.64 | E: ibuprofen |
| E vs. K | -0.15 | 0.84 | -1.46 | 1.00 | 1.31 | 1.30 | 0.32 | 0.63 | F: ibuprofen SUS |
| E vs. M | -1.41 | 0.56 | 0.70 | 1.54 | -2.11 | 1.65 | 0.20 | 0.61 | G: naratriptan |
| F vs. M | -0.97 | 0.80 | -1.30 | 133.03 | 0.32 | 133.03 | 1.00 | 0.64 | H: rizatriptan |
| G vs. M | 0.50 | 0.70 | -1.55 | 126.76 | 2.05 | 126.76 | 0.99 | 0.64 | I: sumatriptan |
| H vs. M | -0.51 | 0.34 | -1.31 | 64.97 | 0.80 | 64.97 | 0.99 | 0.64 | J: sumatriptan NS |
| I vs. M | -0.01 | 0.32 | -1.30 | 54.61 | 1.29 | 54.61 | 0.98 | 0.64 | K:zolmitriptan |
| J vs. M | -1.03 | 0.34 | -1.21 | 53.42 | 0.18 | 53.42 | 1.00 | 0.64 | L: zolmitriptan NS |
| K vs. M | -0.48 | 0.55 | -0.74 | 2.18 | 0.26 | 2.25 | 0.91 | 0.68 | M: placebo |
| **Use of rescue drugs from 2 to 24 hours** | | | | | | | | | |
| A vs. D | -0.43 | 0.73 | -1.08 | 1.90 | 0.65 | 2.07 | 0.75 | 0.24 | A: acetaminophen |
| A vs. L | 0.43 | 0.63 | 1.08 | 2.00 | -0.65 | 2.07 | 0.75 | 0.24 | B: almotriptan |
| B vs. L | 0.59 | 0.45 | 0.38 | 200.03 | 0.22 | 200.04 | 1.00 | 0.23 | C: eletriptan |
| C vs. L | 0.31 | 0.34 | 0.66 | 199.91 | -0.34 | 199.92 | 1.00 | 0.23 | D: ibuprofen |
| D vs. J | -0.44 | 0.98 | -1.10 | 1.92 | 0.65 | 2.08 | 0.75 | 0.24 | E: naratriptan |
| D vs. L | 1.01 | 0.51 | 0.85 | 43.66 | 0.16 | 43.66 | 1.00 | 0.23 | F: rizatriptan |
| E vs. L | 0.10 | 0.40 | 0.87 | 200.06 | -0.76 | 200.06 | 1.00 | 0.23 | G: sumatriptan |
| F vs. L | 0.46 | 0.20 | 0.51 | 115.49 | -0.05 | 115.49 | 1.00 | 0.23 | H: sumatriptan NS |
| G vs. L | 0.32 | 0.23 | 0.65 | 81.56 | -0.33 | 81.56 | 1.00 | 0.23 | I: sumatriptan/naproxen sodium |
| H vs. L | 0.46 | 0.17 | 0.51 | 81.60 | -0.06 | 81.60 | 1.00 | 0.23 | J: zolmitriptan |
| I vs. L | 1.02 | 0.33 | -0.05 | 200.02 | 1.06 | 200.03 | 1.00 | 0.23 | K: zolmitriptan NS |
| J vs. L | 1.64 | 0.88 | 0.98 | 2.07 | 0.65 | 2.08 | 0.75 | 0.24 | L: placebo |
| K vs. L | 0.19 | 0.24 | 0.78 | 141.35 | -0.59 | 141.35 | 1.00 | 0.23 |  |
| **Pain freedom from 2 to 24 hours** | | | | | | | | | |
| A vs. B | 0.33 | 0.47 | 0.05 | 115.69 | 0.28 | 115.69 | 1.00 | 0.25 | A: acetaminophen |
| A vs. E | -0.54 | 0.57 | -1.81 | 2.32 | 1.28 | 2.38 | 0.59 | 0.30 | B: ibuprofen |
| B vs. E | -0.99 | 0.57 | 0.28 | 2.33 | -1.28 | 2.38 | 0.59 | 0.30 | C: rizatriptan |
| C vs. E | -0.55 | 0.30 | -0.65 | 199.83 | 0.10 | 199.83 | 1.00 | 0.25 | D: sumatriptan/naproxen sodium |
| D vs. E | -0.84 | 0.29 | -0.37 | 141.23 | -0.47 | 141.23 | 1.00 | 0.25 | E: placebo |

**eFigure 1:** Overview of risk of bias


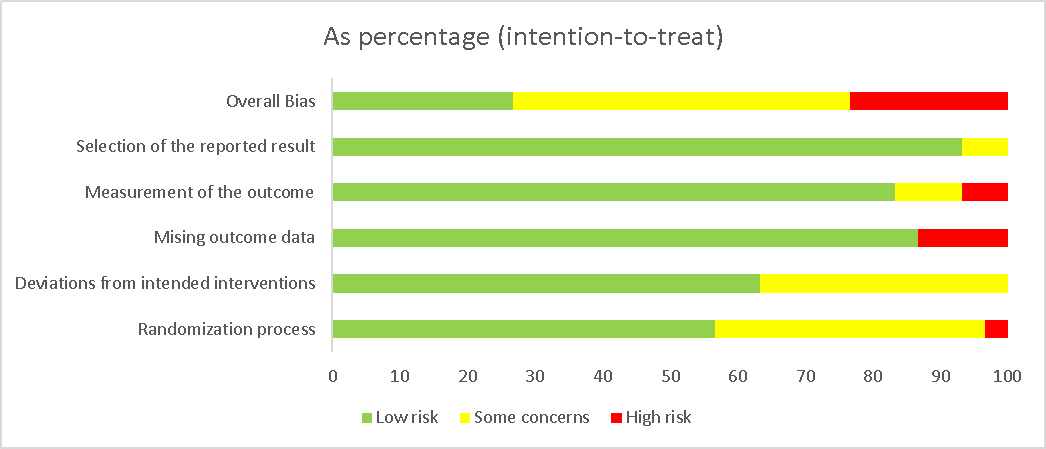


**eFigure 2:** Detailed risk of bias in each study


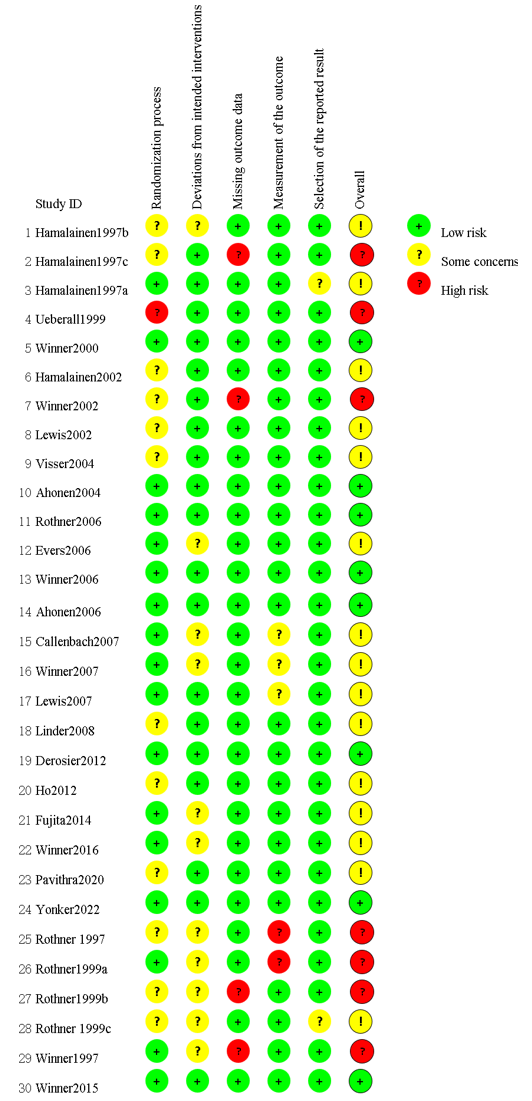


**eFigure 3:** Funnel plot results


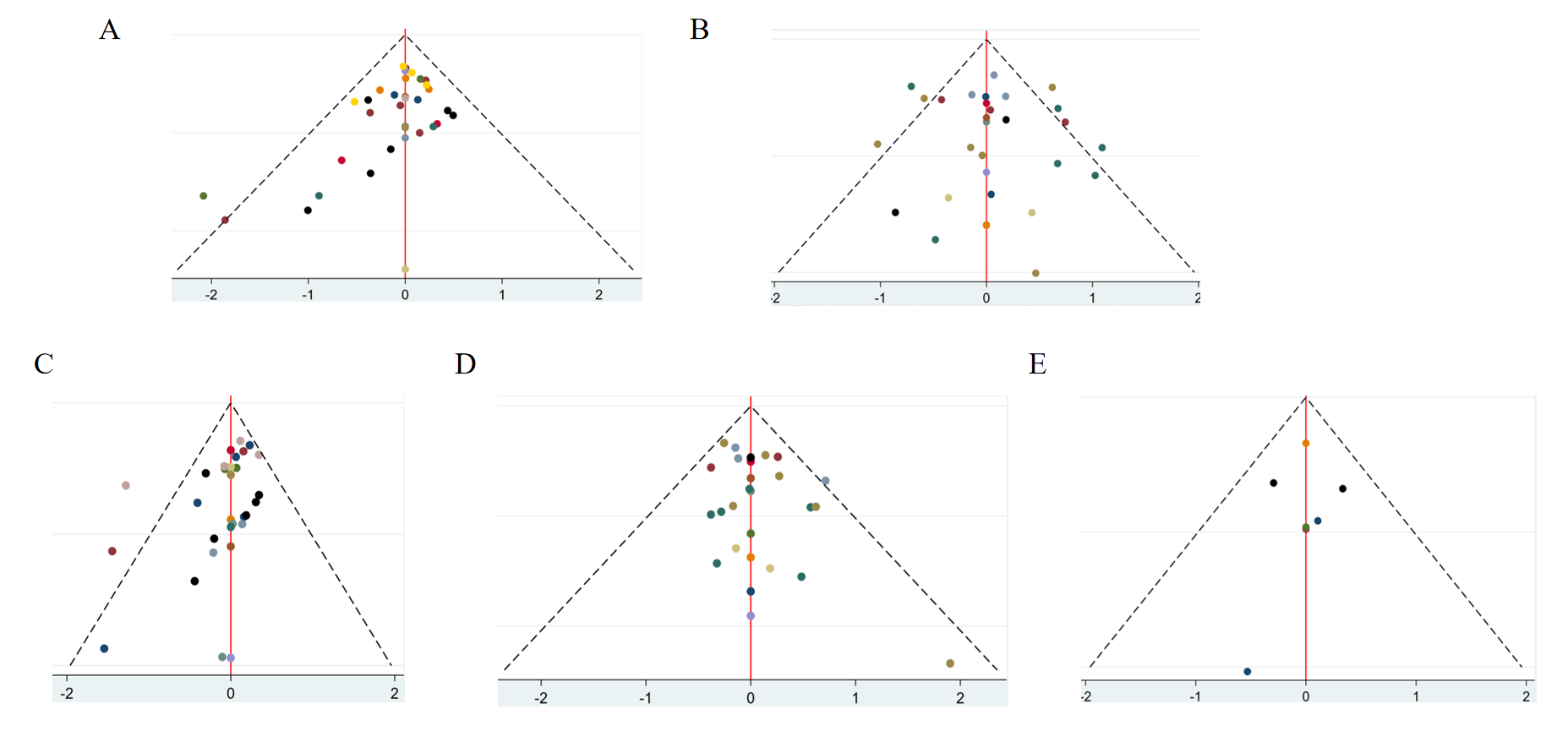


Primary endpoints, (A): pain freedom at 2 hours. (B): adverse events.

Secondary endpoints, (C): pain relief at 2 hours. (D): use of rescue drugs from 2 to 24 hours. (E): pain freedom from two to 24 hours. Egger-value was not reported due to the fewer comparisons available, but the funnel plot was more balanced so we consider the publication bias to be slight.

**References:**

1. Ahonen, K., M. L. Hamalainen, H. Rantala, and K. Hoppu (2004) Nasal sumatriptan is effective in treatment of migraine attacks in children: A randomized trial. Neurology. 62(6): 883-7.

2. Ahonen, K., M. L. Hamalainen, M. Eerola, and K. Hoppu (2006) A randomized trial of rizatriptan in migraine attacks in children. Neurology. 67(7): 1135-40.

3. Callenbach, P. M., L. P. Pels, P. G. Mulder, W. H. Linssen, R. H. Gooskens, J. L. van der Zwan, and O. F. Brouwer (2007) Sumatriptan nasal spray in the acute treatment of migraine in adolescents and children. Eur J Paediatr Neurol. 11(6): 325-30.

4. Derosier, F. J., D. Lewis, A. D. Hershey, P. K. Winner, E. Pearlman, A. D. Rothner, S. L. Linder, D. K. Goodman, T. B. Jimenez, W. K. Granberry, and M. C. Runken (2012) Randomized trial of sumatriptan and naproxen sodium combination in adolescent migraine. Pediatrics. 129(6): e1411-20.

5. Evers, S., A. Rahmann, C. Kraemer, G. Kurlemann, O. Debus, I. W. Husstedt, and A. Frese (2006) Treatment of childhood migraine attacks with oral zolmitriptan and ibuprofen. Neurology. 67(3): 497-9.

6. Fujita, M., K. Sato, H. Nishioka, and F. Sakai (2014) Oral sumatriptan for migraine in children and adolescents: a randomized, multicenter, placebo-controlled, parallel group study. Cephalalgia. 34(5): 365-75.

7. Hämäläinen, M. L., K. Hoppu, E. Valkeila, and P. Santavuori (1997) Ibuprofen or acetaminophen for the acute treatment of migraine in children: a double-blind, randomized, placebo-controlled, crossover study. Neurology. 48(1): 103-7.

8. Hamalainen, M. L., K. Hoppu and P. Santavuori (1997) Sumatriptan for migraine attacks in children: a randomized placebo-controlled study. Do children with migraine respond to oral sumatriptan differently from adults? Neurology. 48(4): 1100-3.

9. Hamalainen, M. L., K. Hoppu and P. R. Santavuori (1997) Oral dihydroergotamine for therapy-resistant migraine attacks in children. Pediatr Neurol. 16(2): 114-7.

10. Hämäläinen, M., M. Jones, J. Loftus, and J. Saiers (2002) Sumatriptan nasal spray for migraine: a review of studies in patients aged 17 years and younger. Int J Clin Pract. 56(9): 704-9.

11. Ho, T. W., E. Pearlman, D. Lewis, M. Hamalainen, K. Connor, D. Michelson, Y. Zhang, C. Assaid, L. H. Mozley, N. Strickler, R. Bachman, E. Mahoney, C. Lines, and D. J. Hewitt (2012) Efficacy and tolerability of rizatriptan in pediatric migraineurs: results from a randomized, double-blind, placebo-controlled trial using a novel adaptive enrichment design. Cephalalgia. 32(10): 750-65.

12. Lewis, D. W., D. Kellstein, G. Dahl, B. Burke, L. M. Frank, S. Toor, R. S. Northam, L. W. White, and L. Lawson (2002) Children's ibuprofen suspension for the acute treatment of pediatric migraine. Headache. 42(8): 780-6.

13. Lewis, D. W., P. Winner, A. D. Hershey, and W. W. Wasiewski (2007) Efficacy of zolmitriptan nasal spray in adolescent migraine. Pediatrics. 120(2): 390-6.

14. Linder, S. L., N. T. Mathew, R. K. Cady, G. Finlayson, G. Ishkanian, and D. W. Lewis (2008) Efficacy and tolerability of almotriptan in adolescents: a randomized, double-blind, placebo-controlled trial. Headache. 48(9): 1326-36.

15. Rothner A, Edwards K. Kerr L. (1997) Efficacy and safety of naratriptan tablets in adolescent migraine. Journal of Neurological Sciences. 150(Suppl 1): S106.

16. Rothner D, Asgharnejad M. (1999) Tolerability of sumatriptan tablets in the acute treatment of migraine in adolescent patients: a review of data from clinical trials. European Journal of Neurology.6(Suppl 3):106.

17. SUMB2003. A double-blind, randomised, placebo-controlled study to compare the eK icacy and safety of oral sumatriptan(25mg, 50mg and 100mg) in the acute treatment of migraine in adolescents. <http://www.gsk-clinicalstudyregister.com/study/SUMB2003#rs.>

18. Rothner D, Asgharnejad M. (1999) Tolerability of sumatriptan tablets in the acute treatment of migraine in adolescent patients: a review of data from clinical trials. European Journal of Neurology.6(Suppl 3):106. [GSK ID: S2CT40].

19. Rothner, A. D., W. Wasiewski, P. Winner, D. Lewis, and J. Stankowski (2006) Zolmitriptan oral tablet in migraine treatment: high placebo responses in adolescents. Headache. 46(1): 101-9.

20. Ueberall, M. A. and D. Wenzel (1999) Intranasal sumatriptan for the acute treatment of migraine in children. Neurology. 52(7): 1507-10.

21. Visser, W. H., P. Winner, K. Strohmaier, M. Klipfel, Y. Peng, K. Mccarroll, R. Cady, D. Lewis, and R. Nett (2004) Rizatriptan 5 mg for the acute treatment of migraine in adolescents: results from a double-blind, single-attack study and two open-label, multiple-attack studies. Headache. 44(9): 891-9.

1. Winner P, Prensky A, Linder S, DeBussey S, Asgharnejad M. Adolescent migraine: efficacy and safety of sumatriptan tablets. Journal of the Neurological Sciences 1997;150(Suppl 1): S172.

23. Winner, P., A. D. Rothner, J. Saper, R. Nett, M. Asgharnejad, A. Laurenza, R. Austin, and M. Peykamian (2000) A randomized, double-blind, placebo-controlled study of sumatriptan nasal spray in the treatment of acute migraine in adolescents. Pediatrics. 106(5): 989-97.

24. Winner, P., D. Lewis, W. H. Visser, K. Jiang, S. Ahrens, and J. K. Evans (2002) Rizatriptan 5 mg for the acute treatment of migraine in adolescents: a randomized, double-blind, placebo-controlled study. Headache. 42(1): 49-55.

25. A double-blind, placebo-controlled, parallel group study to evaluate two dose levels (5mg And 20mg) of sumatriptan nasal spray in the acute treatment of a single migraine attack in adolescent migraineurs (12-17 years of age). <http://www.gsk-clinicalstudyregister.com/study/SUM30045#rs.>

26. Pitman V. EK icacy, safety and tolerability of oral eletriptan (40mg) for the treatment of migraine in adolescents (12-17 years). Headache 2000;40(5):424-5.

27. Winner, P., S. Linder and A. D. Hershey (2015) Consistency of response to sumatriptan/naproxen sodium in a randomized placebo-controlled, cross-over study for the acute treatment of migraine in adolescence. Headache. 55(4): 519-28.

28. Winner, P., V. Farkas, H. Stillova, B. Woodruff, C. Liss, S. Lillieborg, and S. Raines (2016) Efficacy and tolerability of zolmitriptan nasal spray for the treatment of acute migraine in adolescents: Results of a randomized, double-blind, multi-center, parallel-group study (TEENZ). Headache. 56(7): 1107-19.

29. Pavithra, V., D. Mishra, S. Behera, and M. Juneja (2020) Paracetamol versus Ibuprofen for the Acute Treatment of Migraine Headache in Children: A Blinded Randomized Controlled Trial. Indian J Pediatr. 87(10): 781-786.

[30]. Yonker, M. E., J. Mcvige, L. Zeitlin, and H. Visser (2022) A multicenter, randomized, double-blind, placebo-controlled, crossover trial to evaluate the efficacy and safety of zolmitriptan nasal spray for the acute treatment of migraine in patients aged 6 to 11 years, with an open-label extension. Headache. 62(9): 1207-1217.
